# Assessing the Genetic Influence of Ancient Sociopolitical Structure: Micro-differentiation Patterns in the Population of Asturias (Northern Spain)

**DOI:** 10.1371/journal.pone.0050206

**Published:** 2012-11-27

**Authors:** Antonio F. Pardiñas, Agustín Roca, Eva García-Vazquez, Belén López

**Affiliations:** 1 Departamento de Biología de Organismos y Sistemas, Universidad de Oviedo, Asturias, Spain; 2 Departamento de Biología Funcional, Universidad de Oviedo, Asturias, Spain; University of Utah, United States of America

## Abstract

The human populations of the Iberian Peninsula are the varied result of a complex mixture of cultures throughout history, and are separated by clear social, cultural, linguistic or geographic barriers. The stronger genetic differences between closely related populations occur in the northern third of Spain, a phenomenon commonly known as “micro-differentiation”. It has been argued and discussed how this form of genetic structuring can be related to both the rugged landscape and the ancient societies of Northern Iberia, but this is difficult to test in most regions due to the intense human mobility of previous centuries. Nevertheless, the Spanish autonomous community of Asturias shows a complex history which hints of a certain isolation of its population. This, joined together with a difficult terrain full of deep valleys and steep mountains, makes it suitable for performing a study of genetic structure, based on mitochondrial DNA and Y-Chromosome markers. Our analyses do not only show that there are micro-differentiation patterns inside the Asturian territory, but that these patterns are strikingly similar between both uniparental markers. The inference of barriers to gene flow also indicates that Asturian populations from the coastal north and the mountainous south seem to be relatively isolated from the rest of the territory. These findings are discussed in light of historic and geographic data and, coupled with previous evidence, show that the origin of the current genetic patterning might indeed lie in Roman and Pre-Roman sociopolitical divisions.

## Introduction

The Iberian Peninsula, also called by its ancient Greek name “Iberia”, is a crossroads between mainland Europe, northern Africa, the Atlantic Ocean and the Mediterranean Sea [1]. Its current population is the result of a complex mixture of cultures throughout history, and while it shows similar genetic patterns to the rest of Western Europe, it has been recently recognized as particularly diverse [2]. This diversity is enhanced by its varied geography, in which mountains and rivers are coupled with social and linguistic divisions that favor the existence of barriers to gene flow and isolated populations [3], [4].

The northern third of the Peninsula, which comprises the Spanish autonomous communities of Galicia, Asturias, Cantabria, Basque Country, Navarre, Aragon and Catalonia, is separated from the rest of the territory by the mountains of the central plateau. Even close populations from these communities show distinctive distributions of uniparental genetic markers such as the mitochondrial DNA (mtDNA) and the non-recombining portion of the Y-Chromosome (NRY), a situation that has been defined by some authors with the terms “micro-differentiation” or “micro-geographical differentiation” [5], [6]. This form of genetic structuring, which usually involves small secluded populations [7], [8], might be a consequence of the rugged and difficult landscape of the Iberian north, as it has not been found to date in more southern regions [9], [10]. Nevertheless, even as geography is a common cause of population differentiation, historical and social processes should not be disregarded. In fact, a recent study, which made an in-depth analysis of uniparental lineage patterns in the Basque Country found quite remarkable similarities with the Iron Age tribal divisions of the area [11]. If such relations between social and genetic structures can be distinguished, defining and characterizing them might prove useful for reconstructing the most recent historical and social processes behind the present distribution of genetic lineages in this part of the Iberian Peninsula, which remains one of the most controversial aspects of human phylogeography in the European continent [12]. For studying this matter, is necessary to use populations that display well-defined and long-lasting social and political structures, but which have also been relatively shielded from the important waves of intracontinental migration that have been common in Europe during the last century [13].

Of the aforementioned Spanish communities, Asturias (formally “the Principality of Asturias”) comprises the most mountainous region of Iberia and one of the most mountainous of Atlantic Europe [14]. Traditionally, historians described it as an isolated region, except during a part of the Middle Ages, right after the start of the Reconquista War, when Asturias was the destination of many Christian immigrants from southern Iberian regions. In these times, its influence gradually expanded by laws that granted its inhabitants the possibility of freely populating any idle land they could exploit [15]. Nevertheless, this status declined in the following centuries, after several political changes and conflicts, and by the end of the Medieval period it was already described as a marginal, impoverished province of the Spanish Kingdom [16]. One of the causes of this change was a deeply rooted rural subsistence economy that restrained immigration and favored emigration for many years, until the development of coal mining facilities in the 19^th^–20^th^ centuries [17]. This lack of recent immigration can be appreciated in the distinctive surname patterns of Asturias, which indicate the highest levels of inbreeding of all Spanish regions [18].

**Figure 1:**
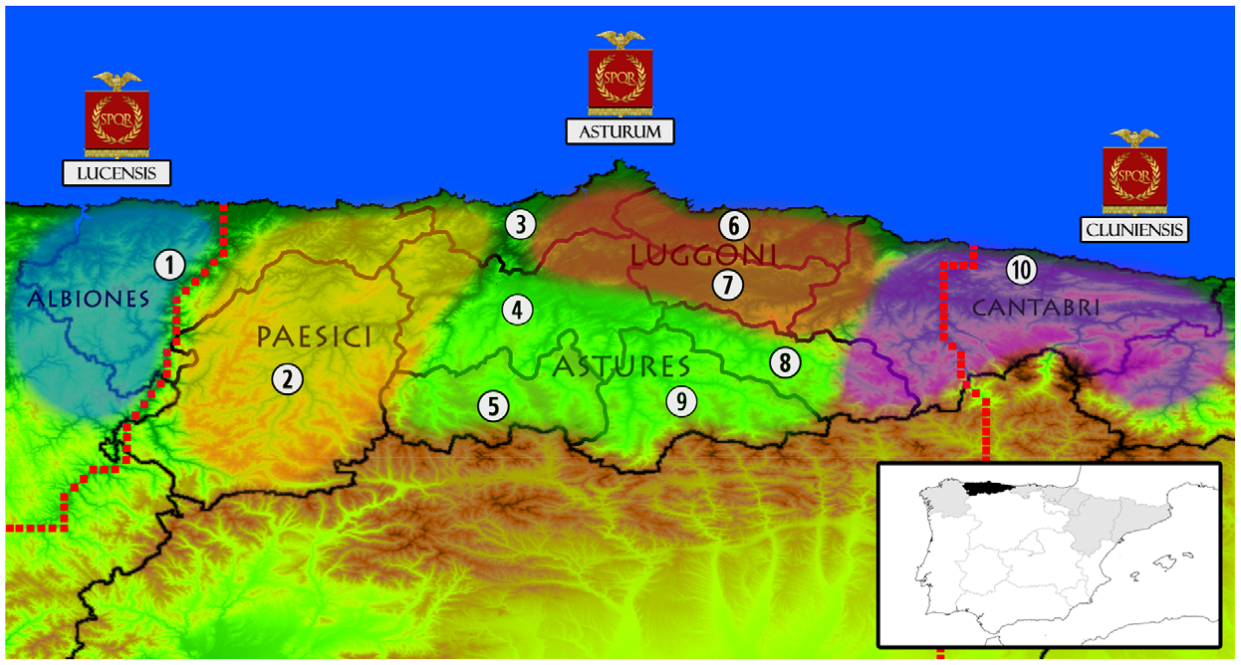
Map of the current territorial division of Asturias, coupled with the political state in the time of the Roman Empire. Numbers and internal lines indicate the regions from which samples were collected in the present study (1: EoNavia; 2: Narcea; 3: Aviles; 4: Central Oviedo; 5: Southern Oviedo; 6: Gijon; 7: Eastern Oviedo; 8: Nalon; 9: Caudal; 10: Oriente). Coloured bubbles represent the known extension of the most important pre-Roman tribes, according to archaeological, historical and epigraphic sources (see text). Names under imperial vexilla represent the Roman subprovincial division *(“conventus iuridicus”*), with its precise borders marked with a red dotted line. Also, a small political map of Iberia is shown in the lower right. Asturias is shaded in black while other communities on the northern third of the Peninsula are shaded in gray.

The land area of Asturias, of around 10.000 square kilometers, has not changed much since it was demarcated by the Roman Emperor Vespasian in 73 CE [19]. The complex orography of its interior, with numerous rivers and mountain ranges, favored the existence of differentiated population groups throughout its history. The oldest of these from which there is a reliable knowledge are the Celtic peoples whose society and customs were thoroughly described by Roman chroniclers such as Strabo of Amasia, Pliny the Elder and Pomponius Mela. Their tribal limits and ranges have been recently defined by a combination of these accounts with other archaeological, historic and epigraphic data (see [20] for a review). While a high number of clans are described in Roman chronicles, sometimes in a contradictory manner, all of these were part of the five major tribes which shared the Asturian territory, demarcating their borders on the basis of geographical features, some of which were later used by the Roman political authority (Figure 1). After the fall of the Roman Empire in the 5^th^ century, it is unclear how the social organization evolved in Asturias, and the ensuing internal division that is well-known is that of the Medieval Feudal and Monastic lordships [21]. These were integrated into a central administration during the 16^th^ century, and are the origin of the current eight territorial regions in which Asturias is politically divided, as defined by the Decree for the Asturian Territorial Planning (Law 11/1991–24^th^ January).

The availability of such a long lasting internal division, coupled with the difficult terrain and the proposed tendency to isolation, makes this region suitable for assessing not only the possibility of micro-differentiation, but also for comparing genetic and sociopolitical structuring in the aforementioned manner. In fact, some evidence of regional differences was already found in classical anthropological studies of the Asturian population based on blood groups and dermatogliphics [22], [23], but their resolution is unsuitable for modern standards. Furthermore, sex-biased social customs common in ancient European populations, such as matrilocality or patrilocality, can be added to the assessment and studied by using uniparental markers [24], [25]. Thus, we will use an in-depth genetic survey of Asturias, based on mtDNA and NRY, for establishing the existence and degree of genetic structuring inside Asturias. Our departure hypothesis is that if this structuring exists, it should be possible to relate it to known geographic and social barriers to gene flow.

## Materials and Methods

### Sampling

Samples of buccal cells were obtained from volunteers recruited in various cities and towns of Asturias during the period 2009–2011 using swabs with Dacron tips (Deltalab; Barcelona, Spain). All female volunteers confirmed having at least two generations of autochthonous maternal ancestry (i.e. grandmothers born in Asturias), while male volunteers confirmed having at least two generations of autochthonous maternal and/or paternal ancestry (i.e. grandmothers and/or grandfathers born in Asturias). After sample collection, volunteers from both sexes with suitable maternal ancestry were used for the mtDNA study (361), while Y-Chromosome was analyzed only for males with suitable paternal ancestry (184). A protocol based on the Chelex-100 resin (Bio-Rad; California, USA) was used for DNA extraction for all samples [26].

### Ethics Statement

The study was approved by the Research and Ethics Committees of the Central University Hospital of Asturias and the University of Leon (Spain) and a written informed consent was obtained from each sample donor, according to the Spanish Law for Biomedical Research (Law 14/2007– July 3^rd^).

### Sequence Groupings

For all population genetic analyses, the samples were grouped to ten populations (Figure 1), on the basis of their maternal or paternal origin. These populations corresponded to the territorial regions which divide Asturias politically. As the Oviedo region has been historically and geographically diverse, it was split to three parts (central, eastern, southern) according to the most parsimonious organization.

### DNA Amplification

The mitochondrial hypervariable segment I (HVS-I) of the 361 samples used in the present study was PCR-amplified for a previous work [27]. To obtain complete control region sequences, the hypervariable segments II and III (HVS-II/HVS-III) were also sequenced from those samples using primers L00015 and H00599 described by Brandstätter et al. [28]. GoTaq® DNA polymerase (Promega; Madison, USA) was used for all PCRs, while sequencing was carried out in an ABI PRISM 3730×l Genetic Analyzer by the company Macrogen (Seoul, Korea). To account for the possibility of sequence ambiguities due to low quality bases, two independent HVS-II/HVS-III amplifications were performed on each sample.

14 Unique-Event Polymorphisms (UEPs) of the non-recombining portion of the Y-Chromosome of 184 samples were amplified and genotyped using the PCR-RFLP protocols described in Table S1. Also, 13 STRs (DYS19, DYS385I/II, DYS388, DYS389I/II, DYS390, DYS391, DYS392, DYS393, DYS460, DYS461, DYS462) were also amplified from each sample following the protocols by Bosch et al. [29]. GoTaq® DNA polymerase was used for all PCRs, while fragment analysis was carried out in an ABI PRISM 3730×l Genetic Analyzer by the Sequencing Service of the Scientific-Technical Services (SCT) of the University of Oviedo.

### Haplogroup Assignment

An updated haplogroup assignment was performed for all mtDNA samples with the software HaploGrep [30], using the full control region sequence and version 13 of the mtDNA phylogenetic tree as a reference [31].

Haplogroup assignment for NRY samples was performed on the basis of the PCR-typed UEPs, using the most updated phylogenetic tree available [32].

### Population Genetic Analysis

Traditional population genetic statistics were obtained with Arlequin v3.512 [33] and DNAsp v5.10 [34]. For mtDNA data, haplotype and nucleotide diversity statistics [35], [36] were estimated, while the θ parameter was computed according to the total number of mutations (η). Demographic history of the female population was assessed by the Fu Fs test of neutrality [37], which can be used as an indicator of population growth or decline. For NRY data, the mean unbiased diversity level of the loci set was estimated [35], while the θ parameter was computed on its basis [38]. Demographic history of the male population was assessed via the Garza-Williamson index designed for microsatellite loci, sensitive to population reductions caused by genetic bottlenecks or mortality crises [39].

Population differentiation was analyzed by means of a classic F_ST_ matrix, computed separately for each dataset. To assess the possibility of a phylogenetic subdivision between populations [40], we also computed a Φ_ST_ matrix based on Tamura-Nei distances for the mtDNA data [41], and a R_ST_ matrix for NRY haplotypes [42]. Additional full control region data and NRY haplotypes of the neighboring Spanish autonomous communities of Galicia, Cantabria and Castilla-Leon were obtained from the available literature and used for assessing the magnitude of population differentiation outside the Asturian territory (Table S2).

Geographic distribution of the different haplogroups was assessed by a principal component analysis (PCA) of haplogroup frequencies of the different regions performed with PRIMER v6 [43]. Data from neighboring regions of Spain (populations from Galicia, Cantabria and the province of Leon); mainland Europe (France, Germany) and the other side of the Cantabrian Sea (England, Ireland) were also obtained from available literature and included in the analysis (Table S3).

To further analyze the effect of geography on the genetic structure of Asturian populations, the software SAMOVA was used to define groups of genetically and geographically homogeneous populations maximally differenced from each other [44], considering both haploid markers separately. These groupings were later used in an Analysis of Molecular Variance (AMOVA) [45] based on Φ_ST_/R_ST_ distances. For comparison purposes, other AMOVAs based on F_ST_ distances and landscape features of the Asturian territory were also computed.

The existence of barriers to gene flow, which usually is a determinant for the establishment of genetic patterns, was assessed with the software BARRIER v2.2 [46] using the aforementioned Φ_ST_/R_ST_ distance matrices. To test for barrier significance, 100 bootstrap replicates of each dataset were computed in MATLAB v7.8 [47] using custom code.

Finally, a rooted and dated tree of population splits was computed using BATWING [48] on the NRY data. Groups inferred by the SAMOVA approach were considered as the population structure for the dataset, under a model of exponential growth and splitting from a constant size ancestral population. A Markov chain of 4 million steps was computed, and final parameter values were taken from the last million steps. Due to the similarities on the analyzed population and the used loci, prior values for the Monte-Carlo Markov Chain (MCMC) computations were taken from the study of Martinez-Cruz et al. [11].

## Results

### Mitochondrial DNA Analysis

#### Genetic diversity and differentiation of Asturian regions

Geographic maternal origin of all samples is reported in Table S4 along with their HVS-II/HVS-III differences to the revised Cambridge Reference Sequence (rCRS) [49]. In the 361 samples, 299 different haplotypes appeared, and 263 of them were singletons. The most common haplotype had the motif 16235G-16291T-93G-263G-309.1C and was found in six samples encompassing four of the ten populations.

Data on census population size and territorial extension of all populations is reported on Table 1 along with their diversity indexes. Genetic diversity values ranged between 1 and 0.972. Additional statistics for each population are given in Table S5. Fu Fs index was significant and negative in almost all cases, a signal of demographic expansion which is currently found in many populations worldwide [50], [51]. The finding of a non-significant but positive value for the populations of Southern and Eastern Oviedo might be a consequence of their limited sample size, as a recent study found that this index can be inflated when computed from a low number of samples, at least in human mtDNA [52]. Nevertheless, we do not expect this situation to bias our other results involving these populations, as those are based in the estimation of F_ST_-analogues which already include corrections for unequal and small sample sizes [53].

**Table 1:** Population data and diversity indexes obtained in the Asturian subpopulations, based on the analysis of mtDNA hypervariable segments and NRY haplotypes.

Population	N	A	n(mt)	K(mt)	H(mt)	π	n(y)	K(y)	H(y)
Aviles	154,627	555	26	25	0.997	0.008	15	15	0.48205
Caudal	67,820	837	44	44	1	0.008	13	13	0.59961
EoNavia	44,211	1,642	54	46	0.99	0.007	31	31	0.51944
Gijón	303,484	525	31	30	0.998	0.008	13	13	0.54931
Nalón	79,842	646	26	25	0.997	0.009	19	19	0.63518
Narcea	29,968	2,127	24	22	0.993	0.008	10	10	0.59487
Oriente	53,386	1,927	81	69	0.994	0.007	43	43	0.57833
Oviedo (Central)	262,372	1,265	48	38	0.981	0.006	27	27	0.56125
Oviedo (South)	4,647	669	9	8	0.972	0.005	3	3	0.79487
Oviedo (East)	67,423	409	19	16	0.982	0.006	10	10	0.59658

N: Region census size.

A: Region area (in km^2^).

n (mt): Sample size of mtDNA sequences.

K (mt): Number of mitochondrial lineages.

H (mt): Haplotype diversity.

π: Nucleotide diversity.

n (y): Sample size of NRY haplotypes.

K (y): Number of NRY haplotypes.

H (y): Nei unbiased diversity measure.

Differentiation between populations was assessed by the comparison of their Φ_ST_/F_ST_ matrices, both shown in Table 2. Most of the pairwise comparisons between Asturian regions didn’t show significant values in any of the statistics (p>0.05). However, when these values did appear, they were more common in the F_ST_ matrix, especially in pairwise comparisons involving the populations of Oriente and EoNavia. Equivalent values in the Φ_ST_ matrix were zero or non-significant, thus indicating an uncoupling between haplotype and molecular differences inside the Asturian maternal pool. The same approach was used for the comparative analysis involving neighboring Spanish autonomous communities, which have been found to be substantially differentiated from most Asturian regions (Table S6).

**Table 2:** Pairwise Φ_ST_ values (below diagonal) and pairwise F_ST_ values (above diagonal) for all Asturian populations, calculated from mtDNA data.

	Aviles	Caudal	EoNavia	Gijon	Nalon	Narcea	Oriente	Oviedo (Central)	Oviedo (South)	Oviedo (East)
**Aviles**	−	0	0.001	0	0	0	0.002	0.001	0.009	0.006 *
**Caudal**	0.003	−	0.001	0	0	0	0	0.001	0	0.006 **
**EoNavia**	0.006	0	−	0.001	0.003	0.006	0.002 *	0.003 *	0	0.007 *
**Gijón**	0.022 *	0	0	−	0	0.003	0	0	0	0.002
**Nalón**	0	0	0	0	−	0	0.003 *	0.001	0	0
**Narcea**	0	0	0	0	0.002	−	0	0	0.013 *	0.006 *
**Oriente**	0.026 **	0.011 *	0.004	0.005	0	0.020 *	-	0.003 **	0	0
**Oviedo (Central)**	0.007	0.004	0.001	0	0	0.013	0.007	−	0	0
**Oviedo (South)**	0	0.013	0.014	0	0.006	0.012	0.013 *	0.006	−	0.008
**Oviedo (East)**	0.017	0	0	0	0.007	0	0.005	0.008	0	−

a Significance tests were performed with 10,100 permutations.

* = p<0.05.

** = p<0.01.

Underlined values are statistically significant**^a^**.

SAMOVA computations revealed maximum differentiation between groups of populations when six geographic groups were formed and the Φ_ST_ measure was used, obtaining an among-group variation of 0.99% (Table 3). These groups have some similarities to the pre-Roman tribal organization, as can be noted in the mtDNA separation between southern and northern central regions (notice the border between the Astures and the Luggoni in Figure 1). This variation is not found if only haplotype composition is regarded, as the among-group differentiation index based on F_ST_ values was not statistically significant. Groups defined purely by geographic features, such as coastal vs. inland regions or a western-central-eastern division also showed no significant structure in none of the AMOVAs, with among-group values close to zero.

**Table 3:** Analysis of Molecular Variance (AMOVA) for mtDNA control region sequence data, using the Φ_ST_ measure (upper) and classical F_ST_ (lower).

Source of variation	d.f.	Sum of squares	Variance components	% variation	Fixation index	p-value^a^
Among groups	5	28.908	0.045	0.990	0.010	0.029
Among populations within groups	4	14.615	−0.031	−0.700	−0.007	0.915
Within populations	352	1579.634	4.487	99.7	0.002	0.224
Total	361	1623.157	4.501			
**Source of variation**	**d.f.**	**Sum of squares**	**Variance components**	**% variation**	**Fixation index**	**p-value** a
Among groups	5	2.614	0	−0.14	−0.001	0.812
Among populations within groups	4	2.157	0.001	0.31	0.003	0.01
Within populations	352	175.423	0.498	99.83	0.001	0.003
Total	361	180.193	0.499			

a Significance tests were performed with 10,100 permutations.

The genetic structure tested consisted of six groups inferred with the SAMOVA approach. 1: Aviles; 2: Caudal-SouthOviedo-Narcea; 3: EoNavia; 4: Gijon-EastOviedo; 5: Nalon-Oriente: 6: CentralOviedo.

**Figure 2:**
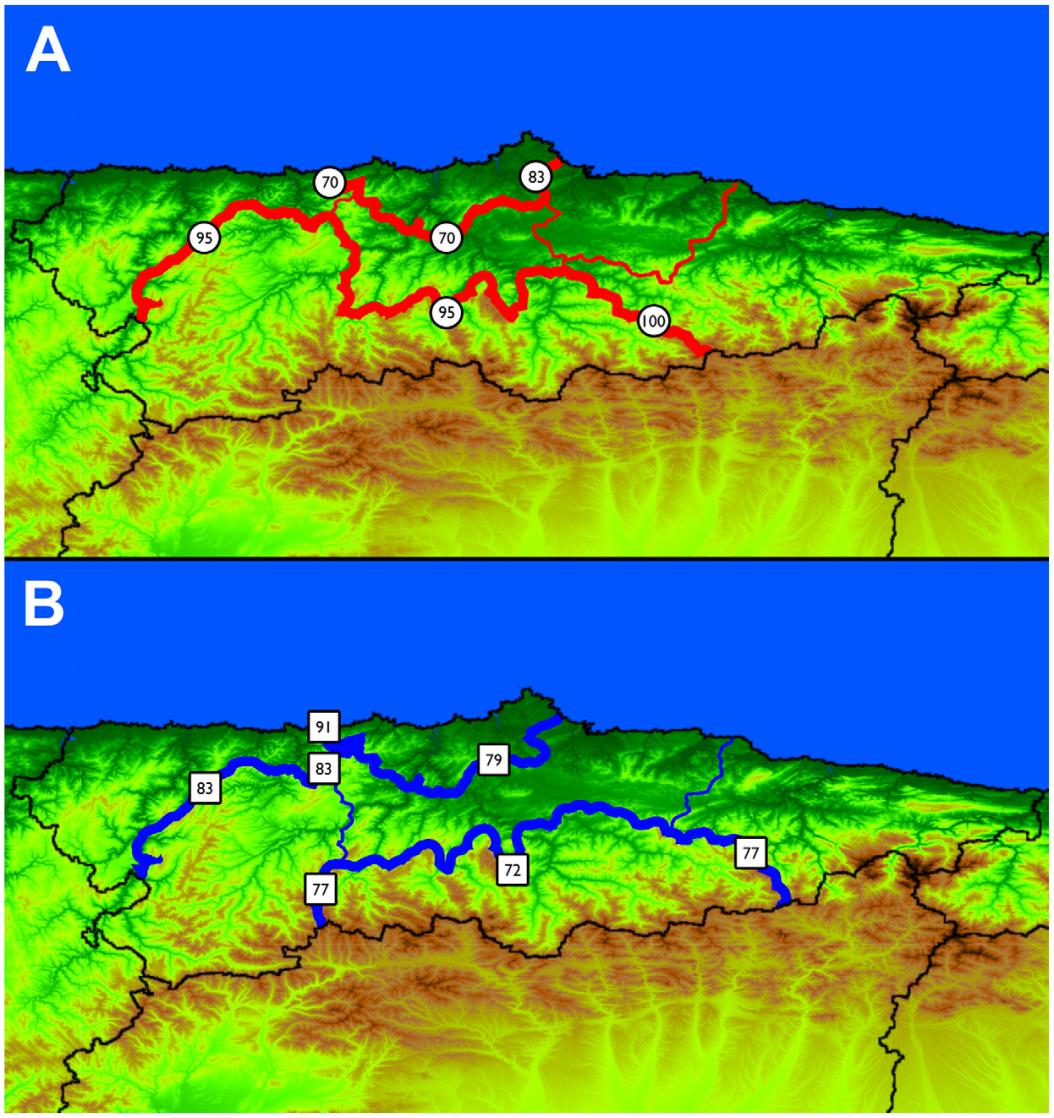
Map of Asturias showing the SAMOVA group division coupled with the inferred barriers to gene flow. Panels show results for the mtDNA data (A) and NRY data (B). Thin lines indicate division in the SAMOVA analysis but no actual barrier inference, while inferred barriers between groups are shown by strong lines. Bootstrap value for each of the barriers is shown next to it and only those with values equal or higher than 70 are shown.

**Figure 3:**
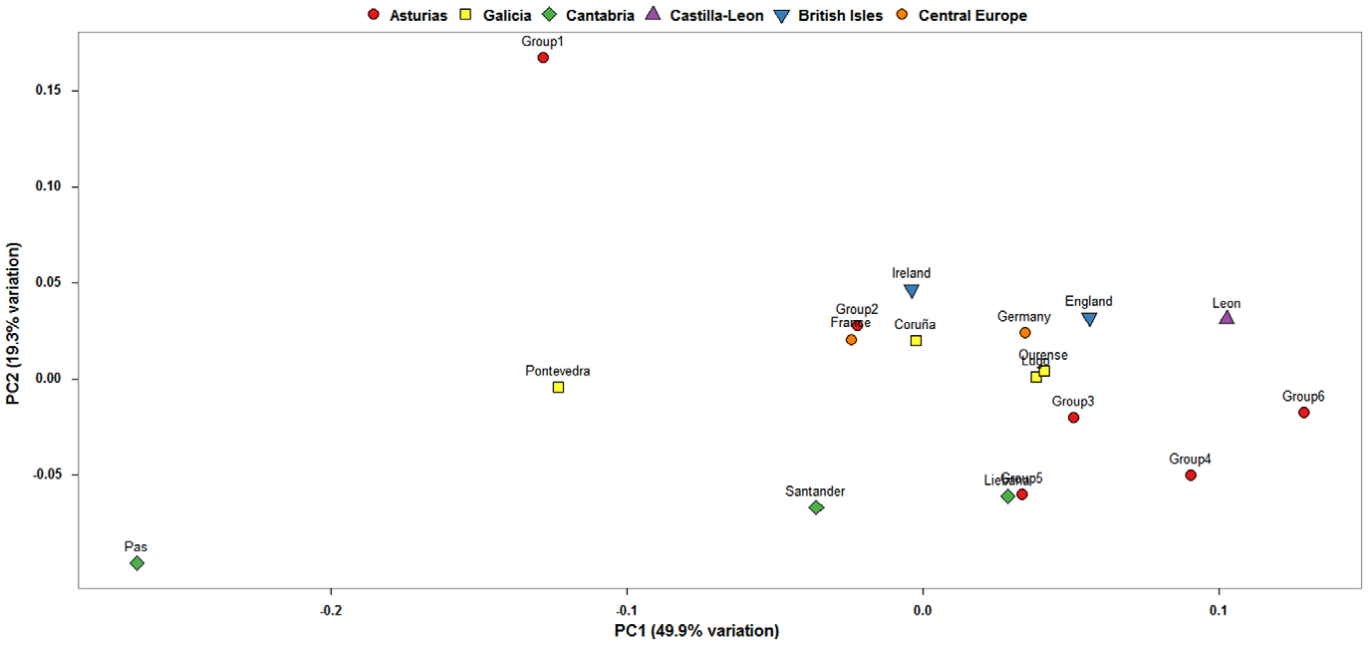
PCA plot of mtDNA haplogroups of Asturias and other regions of Iberia, the British Isles and Mainland Europe.

Results of the BARRIER analysis performed on SAMOVA-inferred groups are shown in Figure 2A. Two barriers to gene flow with high bootstrap values can be highlighted, that completely surround groups 1 and 2. The central part of the region, however, appears as a free corridor from east to west. A PCA plot of haplogroup frequencies of all groups, compared with neighboring regions, is shown in Figure 3. In it, the two groups surrounded by barriers appear deviated from the cluster formed by the other Asturian groups, with group 1 (Aviles region) appearing as an outlier.

### Y-Chromosome Analysis

#### Genetic diversity and differentiation of Asturian regions

Geographic origin of all samples is reported in Table S7 along with their Y-STR motifs. In the 184 samples, 177 different haplotypes appeared, and 171 of them were singletons. The most common haplotype had the motif 13-11-14-12-13-16-24-11-13-13-11-12-11 and was found in three samples from different populations.

Data on census population size and territorial extension of all populations is reported on Table 1 along with their diversity indexes. No haplotypes were found twice inside any population. Unbiased gene diversity values for NRY STR loci ranged between 0.795 and 0.482, with most populations on an intermediate 0.5–0.6 range. Values of the Garza-Williamson index were very close to 1 in all cases, which discards the possibility of recent genetic bottlenecks, supporting the signatures of expansion found in the mtDNA.

Differentiation between populations was assessed by the comparison of their R_ST_/F_ST_ matrices, both shown in Table 4. As expected, molecular distances (R_ST_) are higher than their equivalent haplotype distances (F_ST_) in most pairwise comparisons, but some of those involving the Oriente and EoNavia regions are again showing the opposite trend. The comparative analysis with other neighboring populations follows closely the results obtained for the mtDNA, showing particular NRY pools for each of them (Table S8).

**Table 4:** Pairwise R_ST_ values (below diagonal) and pairwise F_ST_ values (above diagonal) for all Asturian populations, calculated from NRY data.

	Aviles	Caudal	EoNavia	Gijon	Nalon	Narcea	Oriente	Oviedo (Central)	Oviedo (South)	Oviedo (East)
**Aviles**	−	0.043	0.009	0.000	0.040 *	0.058 *	0.022	0.000	0.000	0.044 *
**Caudal**	0.033	−	0.042	0.012	0.010	0.013	0.042 *	0.035 *	0.000	0.014
**EoNavia**	0.027	0.026 *	−	0.000	0.040 *	0.019 *	0.038 *	0.000	0.000	0.019 *
**Gijón**	0.014	0.011	0.000	−	0.057 *	0.023 *	0.024	0.000	0.000	0.000
**Nalón**	0.082 *	0.000	0.033 *	0.010 *	−	0.001	0.043 *	0.022	0.000	0.024 *
**Narcea**	0.066	0.000	0.045 *	0.022 *	0.017	−	0.019	0.000	0.000	0.009
**Oriente**	0.019	0.019 *	0.037 *	0.038 *	0.063 *	0.037 *	−	0.016 *	0.000	0.101 **
**Oviedo (Central)**	0.021	0.000	0.009	0.000	0.019	0.022	0.014	−	0.000	0.000
**Oviedo (South)**	0.000	0.000	0.000	0.000	0.000	0.000	0.000	0.000	−	0.000
**Oviedo (East)**	0.127 *	0.109 *	0.026	0.000	0.133	0.005	0.001	0.006 *	0.000	−

a Significance tests were performed with 10,100 permutations.

* = p<0.05.

** = p<0.01.

Underlined values are statistically significant**^a^**.

SAMOVA computations revealed maximum differentiation between groups of populations when six geographic groups were formed and the R_ST_ measure was used, obtaining an among-group variation of 4.88% (Table 5). Composition of these groups is very similar to that obtained with the mtDNA sequences, and can also be related to the pre-Roman structure. An example can be seen in the differentiation of the Narcea region, historically inhabited by the pastoralist tribe of the Paesici. Nevertheless, differences between NRY groups seem to be deeper than for mtDNA, as an AMOVA based on F_ST_ values was also statistically significant, albeit with a lesser among-group variation of 3.05%. Groups defined by geographic features showed also significant, but minor, among-group variations when tested by the means of an R_ST_ matrix, being 0.77% the result of a coastal vs. inland regions comparison and 0.22% that of a western-central-eastern division. F_ST_ analyses of these groups didn’t show significant genetic structuring.

**Table 5:** Analysis of Molecular Variance (AMOVA) for NRY haplotype data, using the Φ_ST_ measure (upper) and classical F_ST_ (lower).

Source of variation	d.f.	Sum of squares	Variance components	% variation	Fixation index	p-value^a^
Among groups	5	129.901	0.660	4.88	0.048	0.002
Among populations within groups	4	37.769	−0.300	−2.22	−0.023	0.834
Within populations	174	2291.086	13.167	97.34	0.026	0.041
Total	183	2458.755	13.526			
**Source of variation**	**d.f.**	**Sum of squares**	**Variance components**	**% variation**	**Fixation index**	**p-value** a
Among groups	5	31.244	0.114	3.05	0.030	0.004
Among populations within groups	4	12.909	−0.037	−0.99	−0.010	0.858
Within populations	174	641.857	3.689	97.94	0.020	0.006
Total	183	686.011	3.766			

a Significance tests were performed with 10,100 permutations.

The genetic structure tested consisted of six groups inferred with the SAMOVA approach. 1: Aviles; 2: Nalon-Caudal-SouthOviedo; 3: EoNavia; 4: Narcea; 5: Oriente: 6: Gijon-EastOviedo-CentralOviedo.

**Figure 4:**
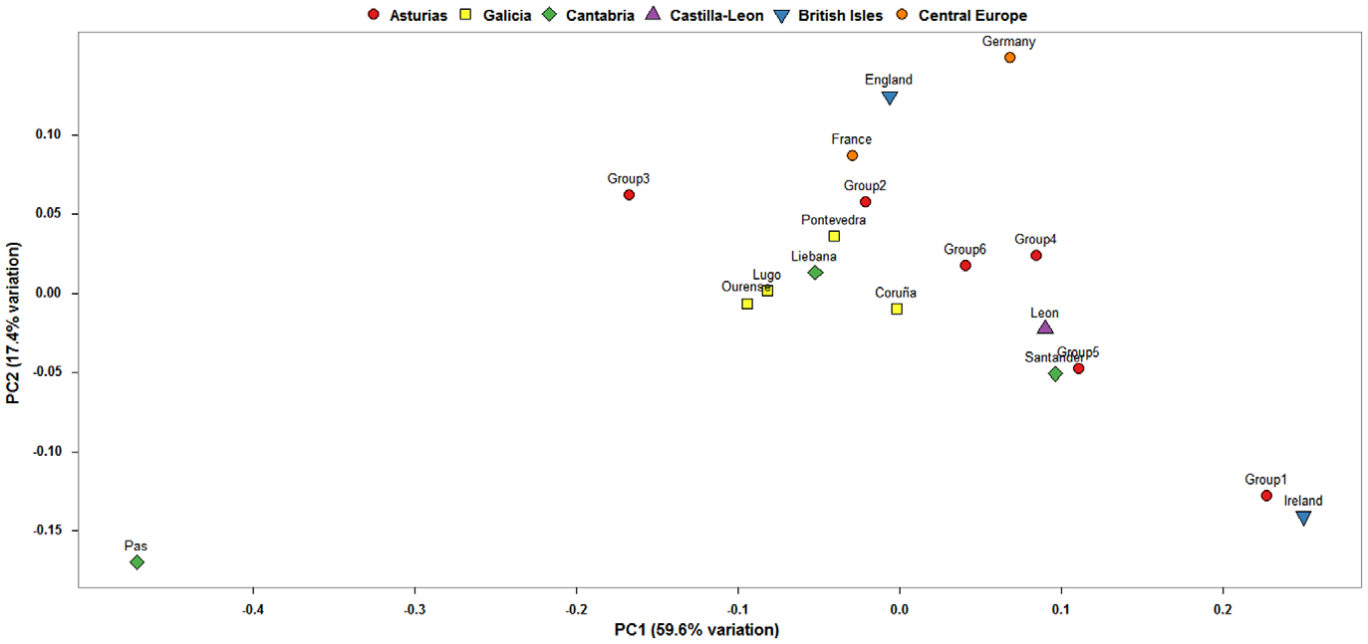
PCA plot of NRY haplogroups of Asturias and other regions of Iberia, the British Isles and Mainland Europe.

Results of the BARRIER analysis are shown in Figure 2B. Again, even as the group composition is not exactly the same, the estimated barriers are analogous to those proposed for the mtDNA data, enclosing the region of Aviles and the southern part of Asturias and leaving a free corridor in the center. In this case, the haplogroup PCA plot (Figure 4) does not appear to have a clear relation with the barriers, but again shows Aviles as an outlier, coupled with Ireland due to their high R1b1a2 haplogroup content.

**Figure 5:**
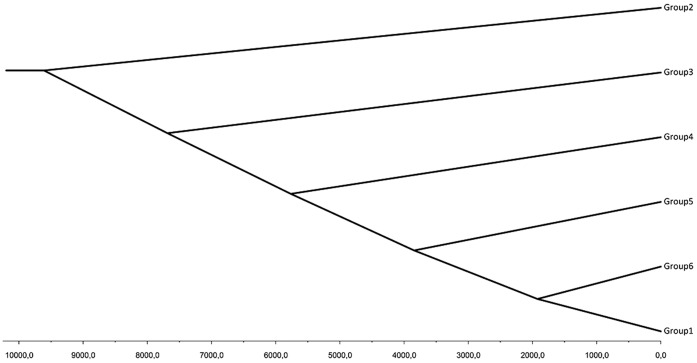
BATWING analysis of SAMOVA-inferred NRY groups. Timescale is shown in years from present. Root of the tree was inferred at 9,609 years ago.

BATWING results are shown in Figure 5 as a cladogram of population splits of the different Y-Chromosome groups. It has to be said that violations of the software assumptions that exist in our dataset (i.e. Admixture between populations) can lead to errors in the molecular dating of the splits, but the sequence of those splits, which was our aim here, is unlikely to be affected, as has been observed in other studies [11].

## Discussion

### Genetic Diversity and Differentiation Patterns Inside Asturias

The Asturian population, as a whole, presents a complex genetic makeup that is highlighted due to the extreme variability of the mitochondrial control region and the NRY microsatellite loci. To obtain an accurate picture of genetic diversity within the Asturian sample, we examined its subpopulations separately. Theoretically, for hypervariable loci such as those used in this study, population differentiation indexes based on haplotype frequencies (F_ST_) should be downwardly biased in respect to indexes based on molecular distances (Φ_ST_/R_ST_), as the frequency of most haplotypes is extremely rare [54]. While this is the occurrence when most Asturian subpopulations are compared, both mtDNA and NRY show the opposite trend in Oriente and EoNavia, respectively the easternmost and westernmost regions. This situation, uncommon in natural populations [40], can be explained by a process of population fragmentation, in which differentiation is based on the recent migration of undiverged haplotypes [55]. This would imply that “historical” populations in the strict sense of the term are not encompassed by current political barriers in Asturias, even though these are related to a territorial division that has spanned several centuries.

SAMOVA analyses of both haploid markers tested in this study are a direct indicator of the genetic structure inside Asturias, and should be a better reflection of population divergence due to the maximum-differentiation criterion used in the algorithm. The mtDNA evidence is remarkable, accounting for the degree of among-group differentiation and the fact that neither among- or within-population indexes are significant. Comparing with other studies that also used full control-region data, these differences are higher than those found between distant Swedish regions [56] and similar to those found between the countries of Greece and Cyprus [57]. Regarding the Y-chromosome, the comparison with Swedish regions is again valid, but the percentage or variation attributable to among-group differentiation is even higher in this case [58]. In fact, this differentiation of Asturian subpopulation groups is similar to that existent between Spanish and Basque Y-Chromosomal pools, which are also affected by a strong micro-differentiation caused by internal barriers [59].

Following previous evidence in the Iberian Peninsula [11], [60], it makes sense to compare the inferred genetic structure to ancient population groups. Finding exact correspondences between contemporary groups and pre-Roman tribes may lead to confounding results, as one of the pacifying strategies that the Roman Empire used in northern Iberia was the forced abandonment of traditional villages and the incorporation of their inhabitants into newly created cities [61]. Nevertheless, this wasn’t always achieved, and in fact the ancient Asturians were labeled as *immunes imperii*, acknowledging that they didn’t recognize Roman rule even though they were militarily subjugated [62]. To facilitate political integration, ancient tribal borders, which followed river basins and other natural features, were recognized by the official Roman territorial administration in many cases. These borders, being both sociopolitical and geographic at the same time, are the best candidates to have been sufficiently long-lasting to leave a mark in both mtDNA and NRY genetic structures. In fact, results of our analyses show a special distinction of the EoNavia and Oriente regions in both haploid markers, whose limits roughly mimic the pre-Roman Albioni and Cantabri tribes. Such tribes are thought to have had their origin in the current territories of Galicia and Cantabria, respectively. Inspection of the PCA plots, which are less sensitive to the high diversity of the Iberian northwest than the pairwise comparisons, reveals that the Oriente region is similar to Cantabrian populations (Liebana in mtDNA, Santander in NRY). Furthermore, it’s the only region in which haplogroup HV4a1a has been found (3.70% of total), which has been recently described as a marker of ancient ascendance from the Franco-Cantabrian refuge [63]. As for the EoNavia region, it shows a more complex position in the PCA plots, being intermediate between Galicia and the rest of Asturias in the mtDNA and occupying a similar position to Oriente in the NRY. It has to be said that part of this region was an important Briton colony after the fall of the Roman Empire [64], which might have contributed with male introgression to its NRY pool.

The Asturian territory is small and the mobility of human populations can establish stable and long-term migratory fluxes over thousands of kilometers [65]. Nevertheless, similar barriers to gene flow were identified with the two types of markers we used (mtDNA and NRY), and in both cases these barriers separated the southernmost group of mountain populations and the region of Aviles from the rest of the groups. It is possible that the ruggedness of the southern terrain, rich in river basins secluded by high mountain ranges (up to 2,000 meters) may have promoted population subdivision and genetic drift in its population. In addition, their differentiation might have been enhanced by the ancestral tradition of consanguine marriages that occurs in some villages of this area, aimed at avoiding dispersal of family lands and possessions [66]. In overall, these facts support and might explain the early population split shown by this group of populations (Group 2) in the BATWING analysis.

Finally, it is difficult to explain the differences between Aviles and the rest of the regions, as the PCA plots show they are of a great magnitude in both markers, and sampling intensity (in relation to the census population) is similar to the other regions. Differences in haplogroup composition are related to the high levels of mitochondrial haplogroup U4 (15%) and Y-chromosomal basal haplogroup F (20%). The first is a typical Eastern European lineage scarce in Southwestern Europe [67], with the exception of the French (but not Spanish) Basques [68]. The other is a Middle Eastern lineage which shows clear peaks in the Caucasus [69] and in some populations of the Mediterranean basin [70]. Looking into the history of Aviles, especially its eponymous main city which houses around 60% of the population of the region, reveals that it became the most important seaport of Asturias during the 12^th^ century onward due to frequent trade with the British Isles, France and Northern Europe [71]. For most of this age, the city was regarded as an important pan-European immigration center, which coupled with a lack of distinctive social or cultural features discards an increased incidence of genetic drift in its population. On the contrary, if increased migration during or after the Middle Ages is responsible for the distinctiveness of this region, it would not need a hindrance of gene flow with other regions to account for the present results, as the inference of such a barrier would be an artifact caused by recent gene introgression. Thus, while there is a low overall NRY diversity in its population, this scenario is supported by the fact that Aviles is the last group to split from the Asturian population in the BATWING analysis.

### Conclusions

This study has combined two highly variable uniparental markers, the mtDNA and the NRY, to assess the diversity, on a regional level, of the population of Asturias. This has allowed us to find, for such a small territory, evidence of micro-differentiation patterns remarkable even in comparison to others found in the Northern third of the Iberian Peninsula. These are explained by considering models of population subdivision based on geographical barriers, but emphasizing the effect of historical events. The resulting structure is compatible with Roman and pre-Roman social and political divisions. While the influences of these are difficult to quantify, their consideration provides a better understanding of genetic data, and of the causes behind its patterning. Further work is needed to characterize and understand the local processes that have took place in such a complex territory and to see if they are can be used to infer demographic and phylogeographic processes at a regional or national scale.

## Supporting Information

Table S1 **PCR-RFLP protocols used for the NRY haplogroup determination.**
(PDF)

Table S2 **Additional genetic data used in the population differentiation analyses.**
(PDF)

Table S3 **Additional haplogroup frequency data used in the PCA plots.**
(PDF)

Table S4 **Geographic and genetic details of samples used in the mtDNA analyses.**
(PDF)

Table S5 **Extended version of** **Table 1** **from manuscript, including effective population estimators and demographic indicators.**
(PDF)

Table S6 **Φ_ST_/F_ST_ matrices showing a comparison of Asturian regions with neighboring populations, based on mtDNA data.**
(PDF)

Table S7 **Geographic and genetic details of samples used in the NRY analyses.**
(PDF)

Table S8 **R_ST_/F_ST_ matrices showing a comparison of Asturian regions with neighboring populations, based on NRY data.**
(PDF)

Reference List S1 **Additional references used in Supporting Information files.**
(PDF)
