# Correction: Assessing the Genetic Influence of Ancient Sociopolitical Structure: Micro-differentiation Patterns in the Population of Asturias (Northern Spain)

**DOI:** 10.1371/annotation/fe924ad4-72bd-47d0-8592-4a942c814520

**Published:** 2013-10-10

**Authors:** Antonio F. Pardiñas, Agustín Roca, Eva García-Vazquez, Belén López

The Supporting Information Table S7 contained an error in the reported repeat-number data for the marker DYS19. Consequently, the motif cited in the text as the most common Y-STR haplotype of Asturias should be 14-11-14-12-13-16-24-11-13-13-11-12-11. The authors regret this and apologize for any inconvenience caused to readers. Please see the corrected Table 7 here: 
