# Supplementary material for: Assessing the Genetic Influence of Ancient Sociopolitical Structure: Micro-differentiation Patterns in the Population of Asturias (Northern Spain)
Source: PLoS One. 2012 Nov 27;7(11):e50206. doi: 10.1371/journal.pone.0050206 (PMC3507697; doi:10.1371/journal.pone.0050206)
Supplement: Table S1 — PCR-RFLP protocols used for the NRY haplogroup determination. (PDF) [file pone.0050206.s001.pdf]

TABLE S1

PCR-RFLP protocols used for the NRY haplogroup determination.

**UEP**	**Detection method**	**Primer pair**	**Anneling T**	**Restriction enzyme**	**Reference**
12f2	PCR (Allele size)	F:CTGACTGATCAAAATGCTTACAGATC<br>R: GGATCCCTTCCTTACACCTTATAC	59°	-	(Cox 2006)
M167	PCR-RFLP	F: AGGTCTTTTTTGCCTTCTTA<br>R: CTGTTCGCTGCTACGAATCT	52°	BsiHKAI	(Veitia et al. 1997)
M17	PCR-RFLP	F:AGAGTTTGTGGTTGCTGGTTGTTACGCG<br>R: TGATGTAGAGACATCTGAAACCCAC	56°	MluI	(Csányi et al. 2008)
M170	Touchdown PCR-RFLP	F: ATGTTTGTTCAAATAATTGCAGC<br>R: CACAACCCACACTGAAAAAC	58° / 48°	NlaIII	(Cox 2006)
M20	PCR/RFLP	F: GATTGGGTGTCCTCAGTGCT<br>R: CACACAACAAGGCACCAT	61°	SspI	(Varzari 2006)
M207	Touchdown PCR-RFLP	F: AGGAAAAATCAGAAGTATCCCTG<br>R: CTGTTCGCTGCTACGAATCT	58° / 48°	DraI	(Cox 2006)
M213	Touchdown PCR-RFLP	F: TATAATCAAGTTACCAATTACTGGC<br>R: TTTTGTAACATTGAATGGCAAA	55° / 46°	NlaIII	(Cox 2006)
M231	PCR-RFLP	F: ATCATGCTACAACCTTCCAGCC<br>R: GGTGGCCAGAGTCTTTCACATC	54°	TaqI	(Rootsi et al. 2006)
M269	PCR-RFLP	F: GGGGAATGATCAGGGTTTGG<br>R: ACTTCTTTTGTGTGCCTTCTGAG	57°	ScrFI	(Csányi et al. 2008)
M52	Touchdown PCR-RFLP	F: TAATACCTATAAGAATATTGCCTGTA<br>R: GCTCAAGATTCATCAGTCAAGTC	63° / 54°	RsaI	(Cox 2006)
M9	PCR-RFLP	F: GCAGCATATAAAACTTTCAGG<br>R: AAAACCTAACTTTGCTCAAGC	58°	HinfI	(Varzari 2006)
M96	PCR-RFLP	F: GTGTAACTTGGAAAACAGGTCTCC<br>R: TCCCAGGGCTACTGTTTTCCC	55°	BseLI	(Cox 2006)
p15	PCR-RFLP	F: CCTCACATGAATAGAGCCAA<br>R: ACTTTCATCTGCCTTCACGC	52°	MluI	(Csányi et al. 2008)
Yap	PCR (Allele size)	F: CAGGGGAAGATAAAGAAATA<br>R: ACTGCTAAAAGGGGATGGAT	51°	-	(Cox 2006)
