# Supplementary material for: Assessing the Genetic Influence of Ancient Sociopolitical Structure: Micro-differentiation Patterns in the Population of Asturias (Northern Spain)
Source: PLoS One. 2012 Nov 27;7(11):e50206. doi: 10.1371/journal.pone.0050206 (PMC3507697; doi:10.1371/journal.pone.0050206)
Supplement: Table S2 — Additional genetic data used in the population differentiation analyses. (PDF) [file pone.0050206.s002.pdf]

TABLE S2

Additional genetic data used in the population differentiation analyses.

Mitochondrial DNA[a]			Y-Chromosome[b]		
**Region**	**Samples**	**Reference**	**Region**	**Samples**	**Reference**
Galicia	32	(Prieto et al. 2011)	Galicia	88	(Adams et al. 2008)
Cantabria	19	(Behar et al. 2012)	Cantabria	104	(Zarrabeitia et al. 2003)
Castilla-Leon	32	(Prieto et al. 2011)	Castilla-Leon	100	(Adams et al. 2008)

[a] Full control region sequences (np. 16024-576).

[b] Haplotypes defined by 9 Y-STRs (DYS19, DYS389a, DYS389B, DYS390, DYS391, DYS392, DYS393, DYS460, DYS461).
