# Supplementary material for: Assessing the Genetic Influence of Ancient Sociopolitical Structure: Micro-differentiation Patterns in the Population of Asturias (Northern Spain)
Source: PLoS One. 2012 Nov 27;7(11):e50206. doi: 10.1371/journal.pone.0050206 (PMC3507697; doi:10.1371/journal.pone.0050206)
Supplement: Table S3 — Additional haplogroup frequency data used in the PCA plots. (PDF) [file pone.0050206.s003.pdf]

TABLE S3

Additional haplogroup frequency data used in the PCA plots.

Mitochondrial DNA			Y-Chromosome		
**Region**	**Samples**	**Reference**	**Region**	**Samples**	**Reference**
Coruna	92	(Álvarez-Iglesias et al. 2009)	Coruna	101	(Brion et al. 2004)
Lugo	90	(Álvarez-Iglesias et al. 2009)	Lugo	95	(Brion et al. 2004)
Orense	38	(Álvarez-Iglesias et al. 2009)	Orense	37	(Brion et al. 2004)
Pontevedra	60	(Álvarez-Iglesias et al. 2009)	Pontevedra	59	(Brion et al. 2004)
Liebana	45	(Álvarez-Iglesias et al. 2009)	Liebana	51	(Brion et al. 2004)
Santander	51	(Álvarez-Iglesias et al. 2009)	Santander	60	(Brion et al. 2004)
Pas	61	(Cardoso et al. 2010)	Pas	50	(Brion et al. 2004)
Leon	63	(Larruga et al. 2001)	Leon	60	(Flores et al. 2004)
England	142	(Helgason et al. 2001)	England	110	(King and Jobling 2009)
Ireland	300	(McEvoy et al. 2004)	Ireland	796	(Moore et al. 2006)
France	551	(Richard et al. 2007)	France	23	(Semino et al. 2000)
Germany	1311	(Richard et al. 2007)	Germany	190	(Capelli et al. 2003)
