# Supplementary material for: Assessing the Genetic Influence of Ancient Sociopolitical Structure: Micro-differentiation Patterns in the Population of Asturias (Northern Spain)
Source: PLoS One. 2012 Nov 27;7(11):e50206. doi: 10.1371/journal.pone.0050206 (PMC3507697; doi:10.1371/journal.pone.0050206)
Supplement: Table S4 — Geographic and genetic details of samples used in the mtDNA analyses. (PDF) [file pone.0050206.s004.pdf]

TABLE S4

Geographic and genetic details of samples used in the mtDNA analyses.

ID	Maternal Origin	HVSII/HVSIII motif	Haplogroup[†]
005	Gijon	263G 309.1C	H9a
007	Narcea	44.1C 72C 150T 263G	V
020	Oviedo (East)	73G 309.1C 309.2C 309.3C	H3d
022	EoNavia	73G 189G 200G 263G 315.1C	L3f1b4a
025	Oviedo (Central)	152C 263G 309.1C	HV4a*
026	Nalon	73G 150T 263G	U5b*
031	Oviedo (Central)	73G 263G	H3d
032	Oviedo (Central)	263G 302C	H2a2*
034	Narcea	72C 195C 263G	HV0
035	Gijon	263G	H2a2*
041	Narcea	73G 263G 309.1C	U5a1*
045	Nalon	263G	H2a2*
046	Caudal	150T 263G	HV12b
050	Aviles	150T 263G	HV12b
051	Oviedo (South)	150T 263G	HV12b
055	Nalon	73G 152C 195C 263G 309.1C 385G 524.1A 524.2C 524.3A 524.4C	T1a
058	Narcea	73G 150T 151T 228A 263G 309.1C	U5b1g
059	Gijon	73G 242T 263G	J1b1a1
067	Oviedo (Central)	73G 150T 152C 263G 295T 315.1C 489C	J2b1a
068	Oviedo (Central)	263G 315.1C 489C 510T 512C 513A	HV*
069	EoNavia	73G 150T 152C 263G 295T	J2b1a
070	Gijon	146C 263G	H1j
071	EoNavia	263G 309.1C 309.2C	H5
075	Gijon	73G 263G 524.1A 524.2C	U5a1b1*
079	Oviedo (Central)	263G	H2a2*
082	Nalon	263G 302C	H2a2*
090	Oviedo (Central)	73G 152C 195C 198T 263G 499A	U4a1d
091	Caudal	150T 263G 315.1C	HV12b
092	Oviedo (Central)	73G 242T 263G	J1b1a1
095	EoNavia	146C 263G 309.1C	HV*
099	Aviles	263G 315.1C	H5
101	Aviles	73G 263G 309.1C	K*
104	EoNavia	152C 263G	HV4b
105	Gijon	73G 185A 263G 408A 462T 482C 489C	J1c1
106	Oviedo (Central)	73G 185A 228A 263G 295T 315.1C 408A 462T 482C 489C	J1c1
107	Caudal	73G 185A 228A 263G 295T	J1c*
111	Aviles	73G 199C 203A 204C 250C 263G 268T	I1a1
112	Aviles	73G 152C 195C 198T 263G 309.1C 499A	U4a1d
113	Aviles	73G 152C 195C 198T 263G 499A	U4a1d
114	Nalon	73G 152C 315.1C 499A	U4a1*
115	Caudal	72C 152C 195C 263G 291del 315.1C	HV0

118	EoNavia	72C 263G 489C	HV0
121	Oriente	152C 263G 291del 315.1C	H3g
125	EoNavia	263G	H2a2*
127	Caudal	263G 315.1C	H*
130	Oviedo (East)	73G 185A 188G 228A 263G 295T	J1c2
132	Oriente	73G 146C 263G 279C 309.1C	T*
133	Nalon	263G 291del 489C	H2a2*
134	Oviedo (East)	73G 263G	T1a
136	Gijon	263G	H2a2*
138	Oviedo (Central)	73G 195C 263G 315.1C 497T 524.1A 524.2C 524.3A 524.4C	K1a*
139	Caudal	150T 239C 263G 310C 315.1C 315.2C 315.3C523del 524del	H6*
140	Narcea	73G 185A 188G 228A 263G 295T 309.1C 312T 524.1A 524.2C	J1c2
142	Oriente	41T 73G 150T 199C 263G 309.1C	T2e1
145	Oviedo (South)	263G 309.1C	H2a2*
146	Oviedo (Central)	152C 263G 315.1C	H3g
147	Nalon	73G 152C 263G 309.1C 497T 524.1A 524.2C	K1a4c
149	EoNavia	73G 185A 228A 263G 295T	J1c*
151	Caudal	73G 195C	X2d
153	Caudal	73G 207A 263G 309.1C	U6*
154	Nalon	207A 263G 309.1C	H*
159	EoNavia	150T 263G 315.1C	HV12b
162	Oviedo (Central)	263G 315.1C	H2a2*
163	Oviedo (Central)	72C 263G 309.1C	HV0
167	EoNavia	263G 477C	H1c*
170	Oviedo (Central)	73G 151T 152C 263G 315.1C 320T	T2b3*
171	Gijon	152C 263G 315.1C	H3g
176	Aviles	263G	H2a2*
178	Narcea	93G 263G	H2a2b1
179	Narcea	263G 315.1C	H3h
182	EoNavia	rCRS	L3x
183	Oviedo (Central)	73G 152C 263G	K1b1a
184	Caudal	73G 150T 152C 263G 295T 315.1C 489C	J2b1a
190	Narcea	73G 150T	T2b*
191	Oviedo (South)	152C 263G 309.1C 385G	H3g
193	Nalon	93G 263G 309.1C	H2a2b1
194	Oriente	73G 195C 225A 226C 263G 309.1C	X2b
195	Nalon	93G 263G 309.1C	H2a2b1
197	Oviedo (South)	73G 263G 309.1C	T1
198	Oviedo (Central)	263G 290d 309.1C	H2a2*
199	Gijon	263G 315.1C	U5b1d
200	Nalon	263G 315.1C	U5b1d
203	EoNavia	73G 263G 310C 315.1C 315.2C	T2b*
204	Aviles	73G 152C 263G 309.1C 315.1C 497T 524.1A 524.2C	K1a4c
206	EoNavia	195C 263G 315.1C 523del 524del	H*
207	Aviles	150T 239C 263G 309.1C	H6*
209	Gijon	73G 263G 309.1C	T2b*

210	Gijon	73G 185A 188G 228A 263G 295T 309.1C 462T 489C 523del 524del	J1c2
211	Narcea	73G 150T 263G 309.1C	T2e*
212	Oriente	73G 263G 282C 309.1C	U8a
213	Caudal	73G 152C 195C 263G 309.1C 524.1A 524.2C	T1a
216	Gijon	73G 263G 315.1C	H1a3
217	Oviedo (Central)	73G 185A 228A 263G 295T 315.1C 408A 462T 482C 489C	J1c1
218	Gijon	263G 315.1C 523del 524del	H2a2*
219	Aviles	73G 185A 228A 263G 295T 309.1C 462T 482C 489C	J1c1
222	Oviedo (Central)	73G 185A 188G 228A 263G 295T 315.1C 462T 489C 523del 524del	J1c2
223	EoNavia	73G 150T 263G 315.1C	T2b*
224	Gijon	263G 309.1C 385G	H2a2*
225	Oriente	263G 315.1C	H2a2*
226	Caudal	152C 239C 263G 309.1C	H6*
227	Oviedo (Central)	73G 200G 263G 309.1C 309.2C 315.1C 523del 524del	L3f1b4a
228	Caudal	73G 263G 309.1C	T2*
229	Caudal	151T 152C 263G 309.1C	H3g
230	Caudal	263G 309.1C	H1j
231	Caudal	73G 263G 309.1C	T2*
233	Caudal	73G 200G 263G 309.1C 309.2C 315.1C 523del 524del	L3f1b4a
234	Caudal	263G 309.1C	H10a1
237	Caudal	73G 263G 309.1C 497T	K1a3a
238	Oviedo (Central)	93G 151T 263G 315.1C	H3f
240	Oviedo (East)	263G 315.1C	H2a2*
241	Nalon	72C 263G 309.1C	V
242	EoNavia	73G 195C 263G 315.1C 489C	D4k
244	Aviles	249del 263G 309.1C	H2a2*
245	Oviedo (Central)	152C 263G 315.1C	H3g
246	Caudal	73G 263G 315.1C	H1a3
247	Caudal	263G 309.1C 456T	H5
248	EoNavia	73G 150T 152C 263G 295T 309.1C 489C	J2b1a
249	EoNavia	263G 309.1C	H2a2*
250	Oviedo (Central)	73G 150T 263G 309.1C 517T	U5b1d
251	Oviedo (Central)	263G 309.1C 523del 524del	H3h
252	Caudal	93G 152C 195C 203A 263G 309.1C	H3f
253	Nalon	55C 57C 152C 263G 309.1C	H15
254	Oviedo (Central)	263G 315.1C 477C 523del 524del	H1c*
255	Oviedo (Central)	263G 309.1C 456T 523del 524del	H5
256	Caudal	44.1C 55C 57C 146C 263G 309.1C 309.2C 315.1C	H15
257	Oviedo (East)	150T 263G 315.1C	HV12b
258	Oviedo (Central)	73G 239C 263G 309.1C	H6a1a1a
259	Narcea	152C 263G 315.1C	H3g
260	Oriente	73G 263G 309.1C	HV4a1a
263	Oriente	263G 309.1C	H2a2*
264	Oviedo (Central)	263G 309.1C 456T 523del 524del	H5
266	EoNavia	73G 195C 196C 215G 263G 315.1C 499A	U4b3

268	Narcea	73G 150T 263G 309.1C	T2e*
272	Nalon	150T 263G 315.1C	HV12b
273	Oviedo (Central)	146C 152C 263G 315.1C 501T	H1h
274	Oviedo (Central)	73G 263G 315.1C	H1a*
275	Oviedo (South)	263G 309.1C	H2a2*
277	Oviedo (East)	73G 94A 150T 263G 309.1C	U5b1d
278	Aviles	93G 263G 309.1C	H2a2b1
282	Oviedo (East)	93G 263G 315.1C 477C 523del 524del	H1c*
283	Oviedo (Central)	93G 263G 309.1C	H2a2b1
284	Oriente	263G 309.1C	H1j
285	EoNavia	152C 263G 310C 315.1C 315.2C	HV6
286	Oviedo (Central)	263G 315.1C 477C 523del 524del	H1c*
287	Gijon	263G 315.1C 477C 523del 524del	H1c*
288	Gijon	152C 263G 279C 315.1C 523del 524del	HV4a*
291	EoNavia	263G 309.1C 523del 524del	H1
293	Caudal	150T 195C 263G 309.1C	HV12b
294	Oviedo (Central)	56.1C 73G 263G 309.1C	T1a
295	EoNavia	57C 73G 263G 309.1C	T1a
296	Gijon	73G 263G 309.1C	U5a1*
298	Gijon	150T 263G 315.1C	HV12b
299	Caudal	73G 207A 263G 315.1C	T2b*
300	Oviedo (South)	263G 309.1C 456T 523del 524del	H5
304	Gijon	239C 263G 309.1C 523del 524del	H6*
307	Aviles	73G 195C 247A 263G 315.1C 499A 524.1A 524.2C 524.3A 524.4C	U4a3
308	Caudal	73G 152C 263G 315.1C 497T	K1a4c
309	EoNavia	146C 150T 263G 309.1C	HV12b
310	EoNavia	73G 150T 263G 309.1C 533G	U5b1f
312	Oviedo (Central)	73G 263G 315.1C	U6*
315	Oviedo (East)	72C 263G 309.1C	HV0
317	Oviedo (Central)	263G 309.1C	H2a2*
318	Gijon	73G 185A 188G 228A 263G 295T 309.1C 462T 489C	J1c2
320	Oviedo (East)	73G 263G 315.1C 315.2C 523del 524del	H3d
321	Oriente	239C 263G 309.1C	H6*
323	Oviedo (East)	73G 150T 315.1C 523del 524del	U5b1b1*
324	Gijon	93G 263G 309.1C	H2a2b1
325	Gijon	309.1C 315.1C	H2a2*
326	Nalon	73G 207A 263G 315.1C	T2b*
327	Oviedo (East)	73G 185A 188G 228A 263G 295T 309.1C 462T 489C 523del 524del	J1c2
328	Gijon	146C 263G 309.1C 389A	H1h
329	EoNavia	263G 315.1C 523del 524del	H2a2*
331	Nalon	239C 263G 309.1C 315.1C	H6*
332	Oviedo (East)	73G 263G 315.1C	U5a1*
333	Gijon	73G 185A 228A 263G 295T 315.1C 462T 489C	J1c*
334	Oviedo (Central)	73G 152C 263G 309.1C 497T	K1a4c
335	Gijon	263G 309.1C	H10a1

336	Oviedo (Central)	263G 309.1C	H2a2*
337	Oviedo (Central)	263G 310C 315.1C 315.2C	H2a2b*
338	Caudal	73G 185A 188G 228A 263G 295T 315.1C 462T 489C 523del 524del	J1c2
340	Oviedo (East)	73G 263G 315.1C 315.2C 523del 524del	H3d
341	Oviedo (East)	73G 263G 523del 524del	H3d
342	Oviedo (Central)	152C 263G 309.1C 523del 524del	H*
343	Nalon	263G 310C 315.1C 315.2C 315.3C	H2a2*
344	Caudal	73G 195C 263G 315.1C 497T 524.1A 524.2C 524.3A 524.4C	K1a*
345	Caudal	73G 263G 309.1C	T2b3a
346	Caudal	225A 263G 315.1C 523del 524del	H2a2*
347	Caudal	73G 189G 200G 263G 315.1C 523del 524del	L3f1b4a
348	Oriente	73G 195C 263G 315.1C 489C	M1*
349	Caudal	73G 228A 263G 295T 315.1C 462T 489C	J1c*
350	Caudal	263G 315.1C 456T	H5
351	Caudal	73G 185A 188G 228A 263G 295T 309.1C 462T 489C 523del 524del	J1c2
352	Oriente	73G 146C 242T 263G 295T 315.1C 340T 462T 489C	J1b1a1
355	Oviedo (Central)	152C 263G	HV4a*
358	EoNavia	263G 309.1C 523del 524del	H2a2*
359	Gijon	73G 150T 152C 263G	J2b1a
360	Gijon	72C 195C 263G 309.1C 524.1A 524.2C	HV0
361	Nalon	73G 263G 309.1C	U5b2a1b
371	Caudal	195C 263G	HV0
379	Oviedo (Central)	93G 204A 263G	H3f
385	Aviles	200G 263G 309.1C 315.1C	H7a1
386	Nalon	55C 57C 152C 263G 309.1C 309.2C 315.1C	H15
396	Nalon	73G 185A 188G 263G	JT
401	Oviedo (East)	73G 152C 195C 263G 309.1C	T1a
404	Oviedo (East)	93G	H2a2b1
405	Oviedo (East)	150T 263G 315.1C	HV12b
409	Oviedo (East)	152C 249G	HV4a*
425	EoNavia	rCRS	H2a2*
426	Gijon	73G 152C 195C 263G	T1a
437	Gijon	73G 185A 188G 228A 263G 295T 315.1C 462T 489C	J1c2
443	Narcea	263G 309.1C	H2a2*
448	Narcea	73G 152C 263G 309.1C	N1b
451	Nalon	73G 152C 199C 204C 207A 250C 263G 315.1C	I2a
454	Oviedo (East)	93G 204A 263G 315.1C	H3f
456	Oriente	239C 263G 309.1C 315.1C 315.2C 315.3C 523del 524del	H2a2*
457	Oviedo (Central)	263G 309.1C	H2a2*
458	Oriente	150T 263G 315.1C	HV12b
459	Nalon	263G 315.1C 477C 523del 524del	H1c*
462	Caudal	73G 263G 309.1C	T2b*
463	Oviedo (Central)	146C 257G 263G 309.1C 315.1C	H1h
466	EoNavia	73G 152C 263G 285T 309.1C 309.2C 315.1C	U1a2
468	Oviedo (Central)	rCRS	H1b

469	EoNavia	73G 199C 204C 250C 263G 315.1C 573.1C	N1e'I
470	Narcea	73G 195C 263G 315.1C 499A	U4*
472	Caudal	73G 152C 195C 204C 263G 309.1C	T1a
473	Caudal	152C 263G 309.1C	H3g
476	Narcea	73G 152C 263G 315.1C 497T	K1a4c
478	Caudal	73G 119C 189G 195C 204C 207A 214G 227G 263G	W1
479	Caudal	239C 263G 309.1C	H2a2*
482	Aviles	263G 309.1C	H2a2*
484	EoNavia	263G 315.1C	H20
486	EoNavia	263G 310C 573.1C	H2a2*
488	EoNavia	195C 263G 315.1C 523del 524del	H*
489	EoNavia	73G 153G 263G 309.1C	T2b*
490	EoNavia	195C 263G 315.1C 523del 524del	H*
491	EoNavia	263G 315.1C	H20
492	EoNavia	73G 263G 315.1C	U5a2
494	EoNavia	73G 263G 315.1C	U5a1b1e
495	EoNavia	44.1C 263G 315.1C 523del 524del	H2a2*
496	EoNavia	263G 315.1C	H2a2*
497	EoNavia	72C 152C 263G 309.1C	V
498	Narcea	73G 152C 195C 200G 263G 309.1C	T*
499	EoNavia	195C 263G 309.1C 523del 524del	H*
500	Oviedo (Central)	73G 143A 152C 182T 195C 263G 309.1C 523del 524del	L2a
501	EoNavia	73G 146C 263G 309.1C 524.1A 524.2C	K2
502	EoNavia	204C 263G 315.1C	H2a2*
503	EoNavia	73G 263G 309.1C	U6a
504	EoNavia	73G 152C 182T 185C 195C 247A 263G 315.1C 357G 523del 524del	L1b
506	EoNavia	73G 185A 263G 295T 315.1C 462T 482C 489C	J1c1
507	Aviles	73G 146C 152C 263G 315.1C	K2a
508	EoNavia	73G 263G 309.1C	U5a1*
509	Narcea	73G 185A 188G 228A 263G 295T 315.1C 462T 489C 523del 524del	J1c2
510	Narcea	263G 315.1C	H2a2*
511	Aviles	263G 309.1C 309.2C	H2a2b*
512	EoNavia	93G 263G 315.1C 456T 523del 524del	H5
513	EoNavia	73G 199C 204C 250C 263G 315.1C 573.1C	I*
514	EoNavia	73G 150T 195C 207A 263G 295T 315.1C 489C 523del 524del	J2a2
516	Narcea	73G 146C 263G 315.1C 572T	K1*
517	Narcea	150T 239C 263G 309.1C	H6*
518	EoNavia	73G 152C 195C 263G 309.1C 524.1A 524.2C	T1a
519	EoNavia	73G 189G 195C 204C 263G 315.1C	W*
520	Narcea	73G 185A 188G 228A 263G 295T 309.1C 462T 489C 523del 524del	J1c2
521	Oviedo (Central)	263G 315.1C 523del 524del	H1x
523	Oriente	72C 263G 309.1C	V
524	Oriente	152C 263G 315.1C	H3g
525	Narcea	73G 152C 263G 310C 315.1C 315.2C 315.3C 524.1A 524.2C 524.3A 524.4C	U5a1a1

526	Gijon	146C 257G 263G 309.1C 477C	H1c3
527	Aviles	73G 263G	T2c*
528	Oriente	73G 214G 263G 309.1C	T2b*
529	Oriente	150T 263G 315.1C	HV12b
530	Oviedo (Central)	73G 152C 263G 315.1C 524T 524.1A 524.2C	K1b1a
531	Oriente	263G 309.1C 456T	H5
532	Nalon	73G 242T 263G 295T 315.1C 462T 489C	J1b1a1
533	Oriente	263G 309.1C 456T	H5
538	Oriente	73G 114T 263G 315.1C 497T	K1a1
539	Oriente	263G 310C 315.1C 315.2C	H2a2*
540	Oriente	73G 185A 263G 295T 315.1C 462T 489C	J1*
542	Oriente	239C 263G 315.1C	H6*
543	Oriente	73G 263G 309.1C 385G	R9*
544	Oriente	73G 195C 257G 263G 315.1C 499A	U4*
546	Oriente	73G 150T 263G 315.1C 573.1C 573.2C	U5b2a1a
547	EoNavia	152C 195C 257G 263G 309.1C 477C	H1c3
548	EoNavia	263G 309.1C	H2a2*
549	Oriente	239C 263G 309.1C	H6*
551	Oriente	263G 315.1C 456T 523del 524del	H5
552	Oriente	73G 150T 195C 263G 315.1C 533G	U5b1f
553	EoNavia	73G 195C 200G 263G 309.1C 466C 489C	M1b1a
554	Oriente	263G 315.1C 315.2C 315.3C	H1f
555	Oriente	263G 309.1C 309.2C 310C 315.1C	H*
556	Oriente	263G 315.1C	H1f
557	Oriente	41T 73G 150T 263G 309.1C	T2e1
558	Oriente	73G 93G 263G 309.1C	H2a2b1
560	Gijon	73G 207A 263G 309.1C	T2b*
561	Oviedo (Central)	152C 263G 309.1C	H3g
563	Gijon	263G 309.1C	H2a2*
565	Oviedo (Central)	152C 263G 309.1C	H3g
566	Caudal	73G 242T 263G 295T 315.1C 462T 489C	J1b1a1
567	Nalon	72C 263G 309.1C	V
568	Oriente	263G 309.1C 564A	H2a2*
570	Oriente	263G 309.1C 316A	H2a2b*
571	Oriente	73G 207A 242T 263G 295T 315.1C 462T 489C	J1b1a1
572	Oriente	73G 207A 242T 263G 295T 315.1C 462T 489C	J1b1a1
573	Oriente	73G 263G 309.1C	HV4a1a
574	Oriente	73G 263G 309.1C	HV4a1a
575	Oriente	73G 263G 309.1C	T2b*
577	Oriente	73G 189G 200G 215G 263G 315.1C 523del 524del	L3f1b4a
579	Oriente	263G 309.1C	H2a2*
580	Gijon	263G 315.1C	H2a2*
583	Oriente	73G 195C 263G 315.1C 497T	K1a3a
584	Oriente	263G 309.1C	H10a1
585	Oriente	239C 263G 309.1C 460C	H6*
587	Nalon	73G 146C 152C 263G 279C 309.1C 523del 524del	T2c1b

590	Oriente	234G 263G 309.1C	H2a2*
591	Oriente	263G 309.1C	H2a2*
592	Oriente	72C 150T 263G 309.1C 508G	HV0
593	Oriente	73G 151T 263G 309.1C	T2b3*
595	Oriente	73G 150T 152C 263G 315.1C	U5b1b1e
596	Nalon	73G 263G 315.1C	H1a3
598	Oriente	73G 152C 263G 309.1C	R9b2
600	Oriente	263G 310C 456T 513A	H5
603	Oviedo (South)	195C 263G 309.1C	H2a5b
604	Oriente	41T 73G 150T 227G 263G 309.1C	T2e1
608	EoNavia	263G 315.1C	H2a2*
609	Oriente	263G 315.1C	H2a2*
610	Oriente	73G 214G 263G 309.1C	T2b*
611	Oriente	73G 150T 152C 195C 215G 263G 295T 319C 489C 513A	J2a1a
612	Oriente	73G 263G 309.1C 340T	H3d
613	Oriente	93G 263G 315.1C 456T 513A	H5
614	Oriente	73G 263G 309.1C	U5a2
615	Oriente	73G 263G 309.1C	T2b*
616	Oriente	263G 315.1C	H10a1
617	Oriente	152C 263G 315.1C	H3g
618	Oriente	263G 315.1C	H*
619	Oriente	263G 309.1C 315.1C	H2a2*
621	Oriente	257G 263G 309.1C 315.1C 315.2C 477C	H1c3
623	Oriente	263G 315.1C	HV13
624	Oriente	72C 195C 263G 309.1C 524.1A 524.2C	HV0
625	Oriente	73G 263G 309.1C	T2b*
626	Oriente	263G 309.1C 316A 564A	H2a2b*
627	Oriente	60A 263G 309.1C	H*
628	Oriente	73G 263G 315.1C 523del 524del	H1a3
630	Oriente	41T 73G 150T 199C 263G 309.1C 389A	T2e1
632	Oriente	263G 315.1C	H3h
633	Oriente	73G 152C 195C 263G 309.1C 499A	U4a1*
636	Oriente	263G 315.1C	H*
637	Oriente	70A 93G 146C 263G 309.1C	H3f
638	Oviedo (Central)	263G 315.1C 508G 573.1C	H2a2*
639	Oriente	263G 309.1C	HV4a*
641	Aviles	73G 263G 309.1C 315.1C 497T	K1a3a
642	Aviles	73G 185A 188G 228A 263G 295T 309.1C 315.1C 462T 489C 523del 524del	J1c2
644	Caudal	263G 315.1C	HV4b
645	Aviles	71.1G 73G 185A 263G 315.1C 523del 524del	T2b*
646	Narcea	73G 152C 263G 309.1C 384G	T1a2a
647	Gijon	93G 195C 263G 309.1C 315.1C	H3f
648	Oviedo (Central)	263G 309.1C 573.1C	T2c*
649	Caudal	73G 150T 263G 309.1C 517T	U5b1d
650	Oviedo (Central)	73G 263G 315.1C	T*
651	Oviedo (South)	93G 263G 309.1C	H2a2b1

652	Oviedo (Central)	146C 263G 309.1C	H1h
655	Narcea	73G 152C 263G 309.1C 497T	K1a4c
656	Nalon	173G 195C 263G 309.1C 321C	T2b*

† For haplogroup inference, HVSI and coding-region information from Pardiñas et al. (2012) were used.
