# Supplementary material for: Assessing the Genetic Influence of Ancient Sociopolitical Structure: Micro-differentiation Patterns in the Population of Asturias (Northern Spain)
Source: PLoS One. 2012 Nov 27;7(11):e50206. doi: 10.1371/journal.pone.0050206 (PMC3507697; doi:10.1371/journal.pone.0050206)
Supplement: Table S5 — Extended version of Table 1 from manuscript, including effective population estimators and demographic indicators. (PDF) [file pone.0050206.s005.pdf]

TABLE S5

Extended version of Table 1 from manuscript, including effective population estimators and demographic indicators.

Population	N	A	n(mt)	K(mt)	H(mt)	π	θ_η	Fs	n(y)	K(y)	H(y)	θ_H	GW
Aviles	154,627	555	26	25	0.997	0.008	0.014	-3.186***	15	15	0.48205	1.50258	0.8998
Caudal	67,820	837	44	44	1	0.008	0.016	-8.923***	13	13	0.59961	1.58265	0.94615
EoNavia	44,211	1,642	54	46	0.99	0.007	0.017	-6.628***	31	31	0.51944	1.50303	0.92051
Gijón	303,484	525	31	30	0.998	0.008	0.015	-5.049***	13	13	0.54931	1.51964	0.91111
Nalón	79,842	646	26	25	0.997	0.009	0.013	-1.676***	19	19	0.63518	1.65771	0.93718
Narcea	29,968	2,127	24	22	0.993	0.008	0.013	-2.628***	10	10	0.59487	1.57469	0.8011
Oriente	53,386	1,927	81	69	0.994	0.007	0.018	-13.708***	43	43	0.57833	1.55032	0.92885
Oviedo (Central)	262,372	1,265	48	38	0.981	0.006	0.015	-3.6592***	27	27	0.56125	1.53047	0.94829
Oviedo (South)	4,647	669	9	8	0.972	0.005	0.007	2.443	3	3	0.79487	2.56653	0.87821
Oviedo (East)	67,423	409	19	16	0.982	0.006	0.01	0.954	10	10	0.59658	1.57752	0.96795

N: Region census size.
A: Region area (in km^2)
n (mt): Sample size of mtDNA sequences.
K (mt): Number of mitochondrial lineages.
H (mt): Haplotype diversity.
π: Nucleotide diversity.

θ_η: Population-mutation rate parameter inferred from the minimum number of mutations.
Fs: Value of the Fu Fs statistic.
n (y): Sample size of NRY haplotypes.
K (y): Number of NRY haplotypes.
H (y): Nei unbiased diversity measure.
θ_H: Population-mutation rate parameter inferred from the expected heterozygosity.
GW: Value of the Garza-Williamson statistic.

Significance tests were based on 10,000 simulations. The original definition of the Fu Fs test notes that it should be considered as significant at the 5% level if the p-value is below 0.02, which is the initial cutoff value that we used.

* = $p < 0.02$ ** = $p < 0.002$ *** = $p < 0.0002$
