# Supplementary material for: Assessing the Genetic Influence of Ancient Sociopolitical Structure: Micro-differentiation Patterns in the Population of Asturias (Northern Spain)
Source: PLoS One. 2012 Nov 27;7(11):e50206. doi: 10.1371/journal.pone.0050206 (PMC3507697; doi:10.1371/journal.pone.0050206)
Supplement: Table S6 — ΦST/FST matrices showing a comparison of Asturian regions with neighboring populations, based on mtDNA data. (PDF) [file pone.0050206.s006.pdf]

TABLE S6

Pairwise Φ_{ST} values (below diagonal) and pairwise F_{ST} values (above diagonal) for all Asturian populations and neighboring Spanish autonomous communities, calculated from mtDNA data. Underlined values are statistically significant [a].

	Aviles	Caudal	EoNavia	Gijon	Nalon	Narcea	Oriente	Oviedo (Central)	Oviedo (South)	Oviedo (East)	Galicia	Cantabria	Castilla-Leon
**Aviles**	-	0	0.001	0	0	0	0.002	0.001	0.009	0.006*	0.002	0.004	0
**Caudal**	0.003	-	0.001	0	0	0	0	0.001	0	0.006**	0.003	0	0
**EoNavia**	0.006	0	-	0.001	0.003	0.006	0.002*	0.003*	0	0.007*	0.008*	0	0.005*
**Gijón**	0.022*	0	0	-	0	0.003	0	0	0	0.002	0	0	0
**Nalón**	0	0	0	0	-	0	0.003*	0.001	0	0	0.003	0	0.001
**Narcea**	0	0	0	0	0.002	-	0	0	0.013*	0.006*	0.005	0	0.002
**Oriente**	0.026**	0.011*	0.004	0.005	0	0.020*	-	0.003**	0	0	0.003*	0	0.002*
**Oviedo (Central)**	0.007	0.004	0.001	0	0	0.013	0.007	-	0	0	0.014**	0	0.011*
**Oviedo (South)**	0	0.013	0.014	0	0.006	0.012	0.013*	0.006	-	0.008	0.012	0	0.009
**Oviedo (East)**	0.017	0	0	0	0.007	0	0.005	0.008	0	-	0.012**	0.012	0.009**
**Galicia**	0.074***	0.062***	0.051***	0.068***	0.058***	0.066***	0.053***	0.080***	0.025	0.038**	-	0.005	0
**Cantabria**	0.009	0.003	0.008	0.001	0.002	0.005	0.019*	0	0	0.010	0.108***	-	0.002
**Castilla-Leon**	0.074***	0.063***	0.055***	0.068***	0.050	0.070***	0.046***	0.073***	0.018	0.049**	0	0.114***	-

[a] Significance tests were performed with 10,100 permutations.

* = $p < 0.05$ ** = $p < 0.01$ *** = $p < 0.001$
