# Supplementary material for: Assessing the Genetic Influence of Ancient Sociopolitical Structure: Micro-differentiation Patterns in the Population of Asturias (Northern Spain)
Source: PLoS One. 2012 Nov 27;7(11):e50206. doi: 10.1371/journal.pone.0050206 (PMC3507697; doi:10.1371/journal.pone.0050206)
Supplement: Table S8 — RST/FST matrices showing a comparison of Asturian regions with neighboring populations, based on NRY data. (PDF) [file pone.0050206.s008.pdf]

TABLE S8

Pairwise R_{ST} values (below diagonal) and pairwise F_{ST} values (above diagonal) for all Asturian populations and neighboring Spanish autonomous communities, calculated from NRY data. Underlined values are statistically significant [a].

	Aviles	Caudal	EoNavia	Gijon	Nalon	Narcea	Oriente	Oviedo (Central)	Oviedo (South)	Oviedo (East)	Galicia	Cantabria	Castilla-Leon
**Aviles**	-	0.055	0.006	0	0.040*	0.082*	0.019	0	0	0	0.073**	0.066**	0.064**
**Caudal**	0.030	-	0.042	0.023	0.018	0.025	0.040*	0.035*	0	0.010	0.058**	0.092***	0.072**
**EoNavia**	0.007	0.012*	-	0	0.034*	0.076*	0.049*	0.013	0	0.028*	0.058***	0.058***	0.054***
**Gijón**	0	0	0	-	0.010*	0.055*	0.028	0	0	0	0.065**	0.069**	0.055**
**Nalón**	0.121**	0.004	0.032*	0.059*	-	0.004	0.039*	0.005	0	0.047*	0.066***	0.101***	0.077***
**Narcea**	0.121*	0	0.082*	0.068*	0.042	-	0.028	0.037	0	0.047	0.096***	0.129***	0.105***
**Oriente**	0.032	0	0.032*	0.041*	0.054*	0.016*	-	0.016*	0	0.003	0.072***	0.077***	0.070***
**Oviedo (Central)**	0.036	0	0.001	0	0.007	0.039	0.016	-	0	0	0.062***	0.070***	0.059***
**Oviedo (South)**	0	0	0	0	0	0	0	0	-	0	0	0	0
**Oviedo (East)**	0	0	0.008	0	0.08	0.056	0.008	0	0	-	0.074***	0.062***	0.064***
**Galicia**	0.103**	0.065*	0.083***	0.079**	0.130***	0.215***	0.100***	0.077***	0	0.116***	-	0.003	0
**Cantabria**	0.095**	0.080**	0.081***	0.082**	0.149***	0.217***	0.102***	0.098***	0	0.110***	0.002	-	0
**Castilla-Leon**	0.103***	0.060*	0.073***	0.076*	0.129***	0.196***	0.096***	0.084***	0	0.120***	0.001	0	-

[a] Significance tests were performed with 10,100 permutations.

* = $p < 0.05$ ** = $p < 0.01$ *** = $p < 0.001$
