# Supplementary material for: Assessing the Genetic Influence of Ancient Sociopolitical Structure: Micro-differentiation Patterns in the Population of Asturias (Northern Spain)
Source: PLoS One. 2012 Nov 27;7(11):e50206. doi: 10.1371/journal.pone.0050206 (PMC3507697; doi:10.1371/journal.pone.0050206)
Supplement: Reference List S1 — Additional references used in Supporting Information files. (PDF) [file pone.0050206.s009.pdf]

REFERENCE LIST S1

Adams SM, Bosch E, Balaresque PL, Ballereau SJ, Lee AC, Arroyo E, López-Parra AM, Aler M, Grifo MSG, and Brion M. 2008. The genetic legacy of religious diversity and intolerance: paternal lineages of Christians, Jews, and Muslims in the Iberian Peninsula. Am J Hum Genet 83(6):725-736.

Álvarez-Iglesias V, Mosquera-Miguel A, Cerezo M, Quintáns B, Zarrabeitia MT, Cuscó I, Lareu MV, García O, Pérez-Jurado L, and Carracedo A. 2009. New population and phylogenetic features of the internal variation within mitochondrial DNA macro-haplogroup R0. PLoS ONE 4(4):e5112.

Behar DM, Harmant C, Manry J, van Oven M, Haak W, Martinez-Cruz B, Salaberria J, Oyharçabal B, Bauduer F, Comas D et al. . 2012. The Basque Paradigm: Genetic Evidence of a Maternal Continuity in the Franco-Cantabrian Region since Pre-Neolithic Times. Am J Hum Genet 90(3):486-493.

Brion M, Quintans B, Zarrabeitia M, Gonzalez-Neira A, Salas A, Lareu V, Tyler-Smith C, and Carracedo A. 2004. Micro-geographical differentiation in Northern Iberia revealed by Y-chromosomal DNA analysis. Gene 329:17-25.

Capelli C, Redhead N, Abernethy JK, Gratrix F, Wilson JF, Moen T, Hervig T, Richards M, Stumpf MPH, and Underhill PA. 2003. A Y chromosome census of the British Isles. Curr Biol 13(11):979-984.

Cardoso S, Zarrabeitia MT, Valverde L, Odriozola A, Alfonso-Sanchez MA, and de Pancorbo MM. 2010. Variability of the entire mitochondrial DNA control region in a human isolate from the Pas Valley (northern Spain). J Forensic Sci 55(5):1196-1201.

Cox MP. 2006. Minimal hierarchical analysis of global human Y-chromosome SNP diversity by PCR-RFLP. Anthropol Sci 114(1):69-74.

Csányi B, Bogácsi-Szabó E, Tömöry G, Czibula Á, Priskin K, Csosz A, Mende B, Langó P, Csete K, and Zsolnai A. 2008. Y-Chromosome Analysis of Ancient Hungarian and Two Modern Hungarian-Speaking Populations from the Carpathian Basin. Ann Hum Genet 72(4):519-534.

Flores C, Maca-Meyer N, González AM, Oefner PJ, Shen P, Pérez JA, Rojas A, Larruga JM, and Underhill PA. 2004. Reduced genetic structure of the Iberian peninsula revealed by Y-chromosome analysis: implications for population demography. Eur J Hum Genet 12(10):855-863.

Helgason A, Hickey E, Goodacre S, Bosnes V, Stefánsson K, Ward R, and Sykes B. 2001. mtDNA and the islands of the North Atlantic: estimating the proportions of Norse and Gaelic ancestry. Am J Hum Genet 68(3):723-737.

King TE, and Jobling MA. 2009. Founders, drift, and infidelity: the relationship between Y chromosome diversity and patrilineal surnames. Mol Biol Evol 26(5):1093.

Larruga JM, Díez F, Pinto FM, Flores C, and González AM. 2001. Mitochondrial DNA characterisation of European isolates: the Maragatos from Spain. Eur J Hum Genet 9(9):708.

McEvoy B, and Bradley DG. 2006. Y-chromosomes and the extent of patrilineal ancestry in Irish surnames. Hum Genet 119(1):212-219.

McEvoy B, Richards M, Forster P, and Bradley DG. 2004. The Longue Duree of genetic ancestry: multiple genetic marker systems and Celtic origins on the Atlantic facade of Europe. Am J Hum Genet 75(4):693-702.

Moore LT, McEvoy B, Cape E, Simms K, and Bradley DG. 2006. A Y-Chromosome Signature of Hegemony in Gaelic Ireland. Am J Hum Genet 78(2):334-338.

Pardiñas A, Roca A, Garcia-Vazquez E, and Lopez B. 2012. Mitochondrial diversity patterns and the Magdalenian resettlement of Europe: New insights from the edge of the Franco-Cantabrian refuge. J Hum Genet DOI:10.1038/jhg.2012.100 [in press].

Prieto L, Zimmermann B, Goios A, Rodriguez-Monge A, Paneto GG, Alves C, Alonso A, Fridman C, Cardoso S, Lima G et al. . 2011. The GHEP-EMPOP collaboration on mtDNA population data--A new resource for forensic casework. Forensic Sci Int Genet 5(2):146–151.

Richard C, Pennarun E, Kivisild T, Tambets K, Tolk H-V, Metspalu E, Reidla M, Chevalier S, Giraudet S, Lauc LB et al. . 2007. An mtDNA perspective of French genetic variation. Ann Hum Biol 34(1):68-79.

Rootsi S, Zhivotovsky LA, Baldovic M, Kayser M, Kutuev IA, Khusainova R, Bermisheva MA, Gubina M, Fedorova SA, Ilumae A-M et al. . 2006. A counter-clockwise northern route of the Y-chromosome haplogroup N from Southeast Asia towards Europe. Eur J Hum Genet 15(2):204-211.

Semino O, Passarino G, Oefner PJ, Lin AA, Arbuzova S, Beckman LE, De Benedictis G, Francalacci P, Kouvatsi A, and Limborska S. 2000. The genetic legacy of Paleolithic Homo sapiens sapiens in extant Europeans: AY chromosome perspective. Science 290(5494):1155-1159.

Varzari A. 2006. Population History of the Dniester-Carpathians: Evidence from Alu Insertion and Y-Chromosome Polymorphisms [PhD thesis]. München, Germany: Ludwig-Maximilians-Universität.

Veitia R, Ion A, Barbaux S, Jobling M, Souleyreau N, Ennis K, Ostrer H, Tosi M, Meo T, and Chibani J. 1997. Mutations and sequence variants in the testis-determining region of the Y chromosome in individuals with a 46, XY female phenotype. Hum Genet 99(5):648-652.

Zarrabeitia M, Riancho J, Gusmão L, Lareu M, Sañudo C, Amorim A, and Carracedo A. 2003. Spanish population data and forensic usefulness of a novel Y-STR set (DYS437, DYS438, DYS439, DYS460, DYS461, GATA A10, GATA C4, GATA H4). Int J Legal Med 117(5):306-311.
