# Supplementary material for: Correction: Assessing the Genetic Influence of Ancient Sociopolitical Structure: Micro-differentiation Patterns in the Population of Asturias (Northern Spain)
Source: PLoS One. 2013 Oct 10;8(10):10.1371/annotation/fe924ad4-72bd-47d0-8592-4a942c814520. doi: 10.1371/annotation/fe924ad4-72bd-47d0-8592-4a942c814520 (PMC3795150; doi:10.1371/annotation/fe924ad4-72bd-47d0-8592-4a942c814520)
Supplement: Supplementary file 1 [file pone.fe924ad4-72bd-47d0-8592-4a942c814520.s001.pdf]

TABLE S7

Geographic and genetic details of samples used in the NRY analyses.

ID	Paternal Origin	Haplogroup	DYS19	DYS385i	DYS385ii	DYS388	DYS389i	DYS389B†	DYS390	DYS391	DYS392	DYS393	DYS460	DYS461	DYS462
001	Aviles	R1b1a2	15	13	18	12	14	16	24	10	13	13	9	11	11
022	EoNavia	E	14	15	17	12	12	18	24	10	11	13	11	12	11
026	Nalon	K	13	13	16	14	12	18	22	10	13	13	10	13	12
040	Oviedo (Central)	R1b1a2	14	10	14	12	12	17	23	11	13	13	11	12	10
049	Nalon	R1b1a2	14	11	14	12	12	17	23	10	13	13	11	12	11
050	Oviedo (Central)	J	14	13	20	16	12	17	23	10	11	12	11	11	10
051	Oviedo (South)	R1b1a2	14	11	14	12	13	15	24	11	13	13	10	11	11
058	Narcea	E	13	13	16	12	16	14	23	10	11	13	12	13	11
069	Oviedo (East)	R1b1a2	14	11	14	12	12	17	24	10	13	13	12	13	11
107	Oviedo (East)	I	14	11	21	12	13	16	24	11	13	12	11	11	11
110	Oviedo (Central)	R1b1a2	14	11	14	12	12	17	24	10	13	13	11	12	11
111	Oviedo (Central)	R1b1a2	14	11	16	12	13	16	24	11	13	13	11	12	11
112	Nalon	R1b1a2	14	11	14	12	12	17	23	11	13	13	11	12	11
113	Gijon	R1b1a2	14	11	14	12	13	16	24	11	13	13	11	12	11
114	Nalon	R1b1a2	14	11	14	12	13	16	24	11	13	13	12	11	10
115	Oviedo (Central)	G2a	16	14	15	13	11	17	22	11	11	14	14	11	12
118	Oviedo (Central)	R1b1a2	14	11	14	12	12	17	23	11	13	13	11	13	10
120	Narcea	R1b1a2	14	11	14	12	13	17	23	11	13	13	12	12	11
122	Oviedo (East)	E	14	16	20	12	14	16	24	10	11	13	12	12	12
124	Nalon	E	14	11	14	12	12	17	24	10	11	13	14	14	11
127	Oviedo (Central)	R1b1a2	14	11	14	12	13	16	24	11	13	13	11	12	11
128	Oviedo (Central)	E	13	17	21	12	13	17	24	9	11	13	11	12	11
132	Gijon	R1b1a2	14	15	21	12	14	17	24	10	13	13	7	12	13

135	EoNavia	R1b1a2	14	11	14	12	13	16	24	10	13	13	11	12	11
136	Gijon	E	13	15	17	12	13	18	23	10	11	13	10	11	12
137	Narcea	J	14	12	17	17	13	16	23	10	11	12	12	13	10
138	Oviedo (East)	R1b1a2	14	11	15	12	13	16	24	10	13	13	12	13	10
140	Oviedo (Central)	R1b1a2	14	11	16	12	14	16	24	11	13	13	11	12	11
144	Nalon	J	14	13	16	12	13	17	24	11	13	13	12	14	11
153	Caudal	R1b1a2	14	11	14	15	13	16	23	10	11	12	11	11	11
154	Oviedo (East)	I	15	12	17	12	12	16	24	11	13	13	11	12	12
155	Nalon	R1b1a2	14	11	14	15	13	18	23	10	11	12	10	11	11
160	Aviles	R1b1a2	14	11	14	12	15	15	23	11	13	13	11	11	11
168	Aviles	R*	14	11	14	12	13	16	24	10	13	13	11	11	11
169	Oviedo (South)	R1b1a2	14	12	14	12	13	16	24	8	13	13	11	12	12
170	Oviedo (East)	F	13	12	16	12	14	16	25	11	13	13	10	11	12
171	Narcea	R1b1a2	14	11	14	12	13	16	24	10	13	13	11	12	11
172	Gijon	R1b1a2	14	12	17	12	13	16	24	11	13	13	12	13	11
180	Aviles	R1b1a2	14	11	14	12	13	16	24	11	13	13	10	11	11
182	Caudal	R1b1a2	15	11	14	12	13	16	26	11	13	13	10	11	11
190	EoNavia	I	14	11	14	12	14	16	24	10	13	13	12	13	12
191	Oviedo (Central)	K	15	13	18	13	12	17	23	10	12	14	10	11	12
195	Nalon	R1b1a2	14	12	14	12	13	17	24	10	13	13	11	12	11
196	Aviles	R1b1a2	14	11	14	12	14	16	24	11	13	13	11	12	11
199	Nalon	K	14	11	12	12	13	16	24	11	13	13	11	12	11
200	Gijon	F	14	12	16	12	12	18	24	10	13	13	10	11	12
208	Oriente	R1b1a2	14	13	16	12	13	16	24	10	13	12	9	10	11
228	Oviedo (Central)	F	14	12	15	14	12	16	23	10	11	13	11	11	12
230	Oriente	E	14	18	21	13	13	17	23	10	11	14	10	10	12
234	Caudal	I	15	12	15	13	12	16	23	10	12	14	11	11	13
237	Caudal	E	13	14	15	12	14	15	21	10	11	13	12	13	13

241	Nalon	J	14	13	19	16	13	18	23	10	11	12	12	12	11
250	Oviedo (Central)	R1b1a2	14	11	15	12	13	16	24	10	13	14	10	11	10
252	Caudal	R1b1a2	14	13	14	12	13	16	24	10	13	13	11	12	11
253	Nalon	R1b1a2	14	11	14	12	12	16	23	11	13	13	11	13	11
259	Oviedo (Central)	R1b1a2	14	11	14	12	14	17	24	10	13	13	11	12	11
267	Oviedo (Central)	R1b1a2	14	12	13	12	13	16	23	11	13	13	12	11	11
269	Aviles	R1b1a2	14	11	13	12	13	16	24	11	13	13	11	12	11
271	Nalon	R*	13	12	13	12	13	16	24	10	13	13	10	11	11
278	EoNavia	F	16	11	14	13	13	16	24	10	11	13	10	11	11
284	EoNavia	R1b1a2	14	12	14	12	13	16	24	10	13	13	11	12	11
288	Oviedo (Central)	J	15	13	17	15	11	17	23	10	11	12	9	10	11
289	EoNavia	R1b1a2	14	11	14	12	14	16	24	10	13	13	11	12	11
291	EoNavia	R1b1a2	14	11	15	12	14	16	24	10	13	13	11	12	11
294	Aviles	R1b1a2	14	11	14	12	13	16	24	11	13	13	11	12	11
296	EoNavia	R1b1a2	14	12	14	12	13	16	23	10	13	13	11	12	11
298	Narcea	R1b1a2	14	11	14	12	13	16	25	11	13	13	11	12	11
303	Nalon	F	15	15	18	13	13	19	22	10	12	14	11	12	12
304	Nalon	J	14	12	16	17	13	18	23	11	11	12	11	12	11
309	EoNavia	R1b1a2	14	11	14	12	14	16	24	10	13	13	11	12	11
311	Caudal	R1b1a2	14	12	14	12	13	16	24	10	13	14	11	13	12
318	Oriente	R1b1a2	14	11	12	12	12	16	24	11	13	13	11	12	11
319	Gijon	R1b1a2	14	12	14	12	13	17	24	11	13	13	11	12	11
322	Narcea	E	13	12	16	12	13	17	21	9	11	13	12	14	12
325	Gijon	R1b1a2	14	12	15	12	14	16	24	10	13	13	10	11	11
327	Oviedo (Central)	R1b1a2	14	14	16	12	13	16	24	11	13	13	7	12	14
330	Gijon	R1b1a2	14	11	13	12	13	16	24	10	15	13	11	12	11
331	Aviles	R1b1a2	14	12	14	12	13	17	24	11	13	13	10	11	11
335	Gijon	R1b1a2	14	11	14	12	13	17	24	10	13	13	10	11	11

340	Caudal	K	14	13	17	17	12	16	23	10	11	12	12	13	11
342	Oviedo (Central)	R1b1a2	14	9	15	12	14	15	24	10	13	13	11	12	11
346	Oriente	R1b1a2	14	11	14	12	13	16	24	11	13	13	11	11	11
348	Oviedo (Central)	R1b1a2	14	11	14	12	13	16	23	11	13	13	11	12	11
351	Caudal	F	15	11	14	15	14	17	23	10	11	12	12	13	11
355	Oviedo (East)	K	14	13	17	12	12	16	25	10	13	13	9	10	11
356	Oviedo (Central)	R1b1a2	14	11	14	12	12	17	23	10	13	13	11	12	11
358	EoNavia	K	15	14	16	12	13	17	23	10	13	13	10	11	12
359	Oviedo (South)	F	15	13	17	15	14	17	23	10	11	12	12	13	12
360	Aviles	R1b1a2	14	11	15	12	13	16	24	11	13	13	12	12	11
378	Nalon	I	17	12	13	13	13	15	23	10	11	13	10	11	12
386	Nalon	J	15	13	14	15	13	17	23	10	11	13	12	13	11
437	Gijon	R1b1a2	13	11	14	12	14	16	24	11	13	13	12	13	11
445	Nalon	R1b1a2	15	11	16	12	13	18	24	11	14	13	11	11	12
451	Gijon	F	16	13	16	13	13	15	23	10	11	13	11	11	13
455	Oviedo (East)	R1b1a2	14	11	15	12	13	16	24	11	13	13	11	11	11
461	Narcea	J	16	12	17	15	13	16	23	10	11	12	12	13	10
465	Gijon	F	15	13	15	13	13	15	23	10	11	13	11	13	13
467	Caudal	R1b1a2	14	13	14	12	13	16	24	10	13	14	11	12	11
471	Caudal	R1b1a2	14	13	14	12	13	16	23	10	13	13	10	11	12
472	Caudal	R1b1a2	14	11	15	12	13	16	25	11	13	13	10	13	11
474	Oriente	R1b1a2	14	12	14	12	13	16	24	11	13	14	10	12	11
478	Oviedo (Central)	E	13	13	15	12	14	16	24	9	11	13	12	13	12
480	Oviedo (Central)	E	15	16	20	12	13	16	24	10	10	13	12	11	12
481	Oviedo (Central)	R1b1a2	14	12	15	12	13	16	24	11	13	13	11	12	11
483	EoNavia	K	15	14	17	12	13	18	23	10	13	13	10	11	12
484	EoNavia	E	13	16	18	12	13	18	24	10	11	13	11	11	11
485	EoNavia	F	16	16	18	13	14	15	23	11	11	13	9	11	11

487	EoNavia	R1b1a2	14	12	13	12	13	16	24	11	13	13	11	12	12
488	EoNavia	R1b1a2	14	11	16	12	14	16	24	11	13	13	11	12	12
489	EoNavia	R1b1a2	14	11	16	12	13	19	24	11	13	13	11	12	11
490	EoNavia	F	15	11	13	13	13	16	23	10	11	13	12	11	12
491	Aviles	F	16	13	18	15	12	15	24	10	11	12	11	10	11
496	EoNavia	E	13	16	18	12	13	19	24	10	11	13	11	11	11
497	EoNavia	E	13	16	18	12	13	18	24	10	11	13	11	12	11
498	Oviedo (Central)	R1b1a2	14	11	15	12	13	17	23	10	13	13	11	12	11
499	EoNavia	R1b1a2	14	11	15	12	13	16	23	11	13	14	11	13	11
502	EoNavia	R1b1a2	14	11	15	12	13	16	24	10	14	13	11	12	11
505	EoNavia	R1b1a2	14	12	13	12	13	15	24	10	13	13	11	11	11
509	Narcea	R1b1a2	14	11	15	12	13	16	25	11	13	13	11	12	11
510	EoNavia	R1b1a2	14	11	16	12	14	18	24	11	13	13	11	12	11
512	EoNavia	R1b1a2	14	11	16	12	13	19	24	11	13	13	11	12	11
513	EoNavia	R1b1a2	15	11	15	12	12	16	23	11	14	13	11	12	11
514	Narcea	R1b1a2	14	11	15	12	12	17	25	11	13	13	11	11	11
515	EoNavia	J	16	12	18	15	13	16	23	10	11	12	11	12	11
517	EoNavia	R1b1a2	14	11	15	12	13	17	24	10	13	13	11	11	11
519	EoNavia	R1b1a2	14	11	16	12	14	18	24	10	14	13	11	11	11
523	EoNavia	J	15	13	16	16	13	16	23	10	11	12	8	12	8
529	Oriente	F	16	12	13	13	14	15	23	10	11	13	12	11	12
531	Oriente	R1b1a2	14	11	16	12	13	16	23	11	13	13	12	11	12
532	Nalon	F	15	13	16	13	13	15	23	10	12	14	12	11	12
533	Oriente	R1b1a2	14	12	15	12	14	16	24	11	13	13	11	11	11
534	Oviedo (Central)	K	14	11	15	12	13	16	24	10	14	13	9	13	11
535	Oriente	E	13	11	15	12	14	16	24	9	11	14	11	12	11
536	Oriente	K	14	11	15	12	14	16	24	11	13	13	11	11	11
537	Oriente	J	15	14	16	15	12	16	25	10	9	12	11	10	11

539	Oriente	R1b1a2	14	11	15	12	13	17	24	10	13	13	9	12	11
541	Oriente	R1b1a2	14	11	15	12	12	16	25	11	13	13	10	12	11
542	Oriente	R1b1a2	14	11	14	12	14	16	25	11	14	13	9	12	11
550	Oriente	R1b1a2	14	11	14	12	13	15	24	11	13	13	11	13	11
552	Oriente	R1b1a2	14	11	15	12	12	16	24	11	13	13	11	11	11
553	EoNavia	E	13	15	17	12	13	17	23	9	11	14	11	11	13
554	Oriente	E	13	11	16	12	14	16	24	9	11	14	11	12	11
557	Oriente	R1b1a2	14	11	15	12	14	15	24	11	13	13	10	12	11
560	Gijon	R1b1a2	14	11	15	12	13	16	24	10	14	13	11	12	11
561	Oviedo (Central)	R1b1a2	14	11	15	12	13	17	24	11	12	13	12	12	12
562	Aviles	F	13	11	15	12	13	16	26	11	13	12	11	12	11
564	Oviedo (East)	R*	15	14	16	12	13	17	23	10	13	13	12	11	12
565	Oviedo (East)	R1b1a2	14	11	15	12	13	16	24	10	13	13	11	12	11
566	Caudal	J	14	12	18	15	13	16	24	9	11	12	11	12	11
569	Oriente	R1b1a2	14	11	14	12	13	16	25	11	13	13	10	11	11
572	Oriente	R1b1a2	14	11	14	12	14	15	23	11	13	13	11	12	11
576	Oriente	R1b1a2	14	12	14	12	11	16	24	12	13	13	11	12	11
577	Oriente	F	14	12	16	15	13	16	22	10	11	12	11	13	11
580	Oriente	F	14	11	14	12	12	16	23	11	13	13	11	12	11
583	Oriente	E	13	12	14	12	14	16	22	9	11	13	12	13	12
587	Oriente	R*	16	11	16	12	12	18	27	11	7	13	12	11	11
588	Oriente	R*	16	11	16	12	12	18	26	10	11	13	12	11	11
590	Oriente	E	13	11	15	12	13	17	22	9	11	13	12	13	12
591	Oriente	R1b1a2	14	11	15	12	13	16	24	12	13	13	12	12	12
593	Oviedo (Central)	F	15	13	15	13	13	15	23	10	12	14	12	12	12
595	Oriente	F	14	12	14	14	12	17	23	10	11	13	12	12	12
596	Oviedo (Central)	R1b1a2	14	11	14	12	14	16	24	11	13	13	11	14	11
598	Oriente	R1b1a2	16	11	15	12	13	18	25	10	9	13	11	11	11

601	Oriente	F	15	13	15	13	12	17	22	10	11	14	12	11	12
604	Oriente	R1b1a2	15	14	17	12	13	16	23	11	14	13	12	11	12
608	Oriente	R*	15	11	16	12	12	18	26	11	11	13	12	11	11
609	Oriente	R*	16	11	16	12	12	18	26	11	11	13	12	11	11
612	Oriente	R1b1a2	15	11	14	12	13	16	25	11	11	13	12	12	12
615	Oriente	R1b1a2	14	11	14	12	13	15	24	11	11	13	11	13	11
617	Oriente	R*	14	11	15	12	14	16	24	10	7	13	12	11	11
618	Oriente	R1b1a2	14	11	14	13	14	16	25	11	13	13	11	12	11
619	Oriente	R1b1a2	14	11	15	12	14	16	22	11	13	13	11	12	11
623	Aviles	F	14	12	15	16	13	18	23	10	11	12	11	12	11
626	Oriente	E	13	12	14	12	14	16	24	9	11	13	12	12	12
627	Oriente	E	13	12	14	12	14	16	24	9	11	13	12	13	12
633	Aviles	R1b1a2	14	12	16	11	14	17	23	10	11	13	9	12	11
635	Nalon	R1b1a2	14	13	16	17	12	18	23	12	11	12	9	12	11
639	Oriente	R1b1a2	14	11	14	12	12	17	24	11	12	14	11	12	11
642	Aviles	R1b1a2	14	11	14	13	14	17	24	10	13	13	11	12	11
643	Oriente	R1b1a2	14	11	15	12	13	17	24	11	13	13	12	12	12
644	Caudal	R1b1a2	14	11	14	12	13	16	24	11	14	13	10	11	11
645	Aviles	R1b1a2	14	11	15	12	13	17	24	11	13	13	10	11	11
652	EoNavia	R1b1a2	15	11	14	12	12	16	26	11	13	13	11	12	11
657	Narcea	E	10	16	20	12	13	18	25	10	11	13	12	12	11

† The repeat length value of DYS389B was calculated by subtracting the value of DYS389I from the value of DYS389II, as in McEvoy and Bradley (2006).
